# A Combined Metagenomics and Metatranscriptomics Approach to Unravel Costa Rican Cocoa Box Fermentation Processes Reveals Yet Unreported Microbial Species and Functionalities

**DOI:** 10.3389/fmicb.2021.641185

**Published:** 2021-02-16

**Authors:** Marko Verce, Jorn Schoonejans, Carlos Hernandez Aguirre, Ramón Molina-Bravo, Luc De Vuyst, Stefan Weckx

**Affiliations:** ^1^Research Group of Industrial Microbiology and Food Biotechnology (IMDO), Faculty of Sciences and Bioengineering Sciences, Vrije Universiteit Brussel, Brussel, Belgium; ^2^LABCIAGRO, School of Agrarian Sciences, Universidad Nacional de Costa Rica, Heredia, Costa Rica; ^3^Laboratory of Molecular Biology, School of Agrarian Sciences, Universidad Nacional de Costa Rica, Heredia, Costa Rica

**Keywords:** metagenomics, metatranscriptomics, cocoa fermentation, microbial diversity, yeasts, lactic acid bacteria, acetic acid bacteria

## Abstract

Cocoa fermentation is the first step in the post-harvest processing chain of cocoa and is important for the removal of the cocoa pulp surrounding the beans and the development of flavor and color precursors. In the present study, metagenomic and metatranscriptomic sequencing were applied to Costa Rican cocoa fermentation processes to unravel the microbial diversity and assess the function and transcription of their genes, thereby increasing the knowledge of this spontaneous fermentation process. Among 97 genera found in these fermentation processes, the major ones were *Acetobacter, Komagataeibacter, Limosilactobacillus*, *Liquorilactobacillus*, *Lactiplantibacillus, Leuconostoc, Paucilactobacillus, Hanseniaspora*, and *Saccharomyces.* The most prominent species were *Limosilactobacillus fermentum*, *Liquorilactobacillus cacaonum*, and *Lactiplantibacillus plantarum* among the LAB, *Acetobacter pasteurianus* and *Acetobacter ghanensis* among the AAB, and *Hanseniaspora opuntiae* and *Saccharomyces cerevisiae* among the yeasts. Consumption of glucose, fructose, and citric acid, and the production of ethanol, lactic acid, acetic acid, and mannitol were linked to the major species through metagenomic binning and the application of metatranscriptomic sequencing. By using this approach, it was also found that *Lacp. plantarum* consumed mannitol and oxidized lactic acid, that *A. pasteurianus* degraded oxalate, and that species such as *Cellvibrio* sp., *Pectobacterium* spp., and *Paucilactobacillus vaccinostercus* could contribute to pectin degradation. The data generated and results presented in this study could enhance the ability to select and develop appropriate starter cultures to steer the cocoa fermentation process toward a desired course.

## Introduction

The raw material needed to produce cocoa liquor or cocoa mass, the basis for the manufacturing of chocolate products, are cocoa beans, the seeds present in the pods of the tropical tree *Theobroma cacao* L. (Schwan and Wheals, 2004; De Vuyst and Weckx, 2016; De Vuyst and Leroy, 2020). Immediately after their harvest, cocoa beans undergo the first steps in the processing chain, namely fermentation and drying. This 4–10 days process mainly takes place in boxes or heaps, usually with periodic mixing, and enables the removal of the pulp surrounding the beans. The fermentation causes biochemical changes within the beans, leading to the formation of flavor and color precursors. Due to their spontaneous nature, cocoa fermentation processes occasionally fail, negatively impacting both the farmers and the chocolate industry.

The pulp contains pectin and high concentrations of glucose, fructose, and citric acid (Afoakwa, 2016; De Vuyst and Weckx, 2016; De Vuyst and Leroy, 2020), presenting a microaerobic environment that favors a typical successive microbial growth during the cocoa fermentation process, beginning with yeasts and lactic acid bacteria (LAB) (Schwan and Wheals, 2004; De Vuyst and Weckx, 2016; De Vuyst and Leroy, 2020). The yeasts consume carbohydrates, produce ethanol, and play prominent roles in aroma formation, as well as the pulp removal through pectin degradation (Schwan et al., 1997; De Vuyst and Weckx, 2016; Samagaci et al., 2016; Meersman et al., 2017; De Vuyst and Leroy, 2020). The LAB consume the carbohydrates and citric acid, produce lactic acid, ethanol, diacetyl, and acetoin, and reduce fructose to mannitol (Lefeber et al., 2010; De Vuyst and Weckx, 2016; De Vuyst and Leroy, 2020). After the increase in ethanol concentration and oxygen accessibility because of pulp liquefaction, acetic acid bacteria (AAB) oxidize the ethanol into acetic acid and, depending on the capacity of the strains present, further into carbon dioxide and water (Lefeber et al., 2010; Moens et al., 2014). Ethanol, acetic acid, and heat produced during the fermentation cause the death of the cocoa bean embryos, resulting in a cascade of events that sets the biochemical stage for a plethora of flavor- and color-forming reactions between peptides, amino acids, carbohydrates, and other compounds, in particular during drying and roasting of the cocoa beans (Voigt et al., 1994; Wollgast and Anklam, 2000; De Vuyst and Weckx, 2016; De Vuyst and Leroy, 2020; Muñoz et al., 2020).

Years of research on the cocoa fermentation process have indicated that yeasts, LAB, and AAB, are key players in these fermentation processes. The resulting focus on these microbial groups is reflected in the use of dedicated growth media during culture-dependent studies that commonly report on the yeast species *Candida tropicalis, Hanseniaspora guilliermondii, Meyerozyma guilliermondii* (formerly *Pichia guilliermondii*), *Pichia kudriavzevii* (formerly *Issatchenkia orientalis*), and *Saccharomyces cerevisiae*; the LAB species *Limosilactobacillus fermentum* (formerly *Lactobacillus fermentum*) and *Lactiplantibacillus plantarum* (formerly *Lactobacillus plantarum*); as well as the AAB species *Acetobacter pasteurianus*, *Acetobacter ghanensis*, and *Acetobacter senegalensis* (Jespersen et al., 2005; Camu et al., 2007, 2008; Nielsen et al., 2007; Daniel et al., 2009; Garcia-Armisen et al., 2010; Papalexandratou and De Vuyst, 2011; Papalexandratou et al., 2011a,b,c, 2013; Lefeber et al., 2012; Crafack et al., 2013; Ho et al., 2014, 2015, 2018; Hamdouche et al., 2015; Meersman et al., 2015; Bortolini et al., 2016; Fernández Maura et al., 2016; Jamili et al., 2016; Koné et al., 2016; Magalhães da Veiga Moreira et al., 2016; Samagaci et al., 2016; Visintin et al., 2016; Balogu and Onyeagba, 2017; de Almeida et al., 2019). Culture-independent studies have extended this knowledge and reported the presence of additional microbial groups, such as enterobacteria and bacilli (Nielsen et al., 2007; Papalexandratou et al., 2011a, 2013, 2019; Illeghems et al., 2012, 2015; Hamdouche et al., 2015; Bortolini et al., 2016; Magalhães da Veiga Moreira et al., 2016; Ouattara et al., 2017; Agyirifo et al., 2019; Serra et al., 2019). However, to show their active involvement, a link should be made between their growth and metabolic activities. Whereas metagenomics can map the microbial species diversity and predict its functional potential (Illeghems et al., 2012, 2015; Agyirifo et al., 2019; Lima et al., 2020), metatranscriptomics can reveal actively transcribed genes that are likely to impact the microbial metabolism. Actual metabolic activities can be assessed through a metabolomics or a metabolite target analysis approach, in particular by measuring the concentrations of predicted metabolites.

The aim of this study was to elucidate the microbial diversity during cocoa fermentation, identify microbial species present therein, and assess their behavior over time by applying metagenomic sequencing on three Costa Rican cocoa fermentation processes. Furthermore, the aim was to ascribe potential functions to microbial groups from a temporal perspective through the combined use of metagenomics, metatranscriptomics, and metabolite target analysis.

## Materials and Methods

### Fermentations and Sampling

Three cocoa fermentation processes (referred to as F1, F2, and F3) were carried out with the Trinitario cocoa variety and sampled on a cocoa farm in the Limón province of Costa Rica in August 2016 (F1) and September 2016 (F2 and F3). Fermentations F2 and F3 were performed in parallel, 2 weeks after the beginning of fermentation F1. Immediately after the cocoa pod harvest, mechanical opening and automated pod removal, the cocoa pulp-bean mass was placed in 1.4 m × 2.0 m × 0.6 m boxes, each containing approximately 1,700 kg. The mass was left to ferment for the first 48 h, followed by daily mixing. The fermentation lasted for 7 days, followed by drying and further processing of the beans. Temperature and pH were monitored on-line by means of a portable digital pH meter (pH 340i; Xylem Analytics, Weilheim, Germany) for fermentations F1 and F2. Samples for RNA extraction (F2 only), DNA extraction, and metabolite target analysis were taken at time points 0 h (when the boxes were full), 7, 20, 44, 68, 92, and 140 h, with a 1 h delay for fermentation F3. For DNA extraction, three cocoa beans with associated pulp (approximately 10 g) were placed into a 50 mL centrifuge tube and stored at −20°C. For RNA extraction, three cocoa beans with associated pulp were placed into 30 mL of RNA*later* (Thermo Fisher Scientific, Waltham, MA, United States). This suspension was mixed and kept refrigerated for 24 h, followed by storage at −20°C. For metabolite target analysis, two samples of approximately 300 g of cocoa pulp-bean mass were stored in freezer bags at −20°C. The samples were transported on ice from the farm to the laboratory at the Universidad Nacional de Costa Rica, Heredia, Costa Rica.

### DNA Extraction

An aliquot of 10 mL of NaCl solution (0.85% m/v) was added to the tubes with the sampled beans and vortexed for 30 s. After removal of the beans, centrifugation at 6,000 × *g* for 10 min, washing the pellet with 2 mL of sorbitol buffer (1.5 M sorbitol, 50 mM Tris, RNase-free water, pH 8.5), and another centrifugation at 6,000 × *g* for 10 min, the pellets were frozen for at least 1 h. After thawing, the pellets were resuspended in 1 mL of lysis buffer [8% (m/v) sucrose, 50 mM ethylenediaminetetraacetic acid (EDTA), 50 mM Tris, 20 mg/mL lysozyme, 125 U mutanolysin, 30 mM β-mercaptoethanol, RNase-free water, pH 8.5]. These suspensions were incubated at 37°C for 45 min, after which 20 μL of Lyticase (20 U/μL stock) and 20 μL of a LongLife Zymolyase solution were added, followed by another incubation at 37°C for 45 min. Then, 0.4 g of acid-washed 0.2 mm glass beads, 100 μL of 20% sodium dodecyl sulfate (SDS), and 100 μL of a proteinase K solution (5 mg/mL) were added to the mixture and the tubes were vortexed for 1 min, followed by an incubation at 56°C for 45 min. One volume of phenol:chloroform:isoamyl alcohol (49.5:49.5:1.0; Sigma-Aldrich, St. Louis, MO, United States) was added to the suspensions, followed by mixing for 1 min and centrifugation at 6,000 × *g* for 10 min. The aqueous phase was transferred to another 15 mL centrifuge tube, to which one volume of AL buffer (Qiagen, Venlo, The Netherlands) and one volume of absolute ethanol were added. The mixture was repeatedly applied to a DNeasy Blood and Tissue column in aliquots of 600 μL until the whole mixture was used, followed by DNA purification with the DNeasy Blood and Tissue Kit (Qiagen), and final elution in 200 μL of AE buffer (Qiagen). After transport to Belgium, an RNase treatment was performed by adding 4 μL of an RNase solution (Thermo Fisher Scientific, Waltham, MA, United States) to 180 μL of the DNA solution and incubating the mixture at 37°C for 30 min, followed by a second DNA purification step using the DNeasy Blood and Tissue Kit.

### RNA Extraction

The tubes containing the cocoa beans in RNA*later* were vortexed for 30 s. The beans were removed from the tubes using sterilized forceps treated with RNase Away (Sigma-Aldrich) and the remaining suspensions were centrifuged at 6,000 × *g* for 30 min. From pellet washing to the incubation with proteinase K, the pellets were processed the same way as described above for the DNA extraction protocol. After centrifugation at 6,000 × *g* for 10 min, the supernatants were transferred to a new 15 mL centrifuge tube, to which 3.5 volumes of RLT buffer (Qiagen) with β-mercaptoethanol and 2.5 volumes of absolute ethanol were added. The mixtures were repeatedly applied to RNeasy Mini columns in aliquots of 650 μL until the whole mixtures were used, followed by RNA purification with the RNeasy Mini Kit according to the manufacturer’s protocol, including on-column DNase treatment (Qiagen). The purity, quality, and integrity of purified RNA were assessed using bleach-agarose gel electrophoresis (Aranda et al., 2012), spectrophotometry (NanoDrop; Thermo Fisher Scientific), and capillary electrophoresis (Bioanalyzer 2100; Agilent Technologies, Santa Clara, CA, United States).

### Metagenomic and Metatranscriptomic Library Preparation and Sequencing

All materials and machines used in this section were from Thermo Fisher Scientific, unless stated otherwise. Metagenomic libraries were prepared from RNA-free DNA samples for all time points of F2 and representative time points of F1 and F3 (7, 20, and 68 h), using the Ion Torrent sequencing platform kits, as described previously (Vermote et al., 2018), with a target fragment length of approximately 300 bp. Thus, 13 metagenomic libraries were prepared. Their names consisted of the notation CRFxDy, whereby CR stood for Costa Rica, Fx represented the fermentation process (F1, F2, or F3), D stood for DNA, and y represented the time point.

Library preparation was followed by template preparation and sequencing using an Ion Torrent PGM, as well as metagenomic data quality control and trimming, as described previously (Vermote et al., 2018). All metagenomic data sets were subjected to quality control and quality trimming with FastQC v0.10.1^1^ and PRINSEQ 0.20.2 (Schmieder and Edwards, 2011). PRINSEQ settings were adapted to each metagenomic data set based on the output of FastQC.

Due to low RNA integrity and the presence of both bacterial and eukaryotic RNA, which prevented universal mRNA enrichment without significant losses, three samples with the highest integrity were chosen for total RNA sequencing, covering three representative time points in fermentation process F2 (7, 20, and 68 h). Library preparation, reverse transcription, and cDNA sequencing were performed at Macrogen (Seoul, South Korea), using a TruSeq Stranded mRNA kit (Illumina, San Diego, CA, United States), a HiSeq 2500 sequencing platform (Illumina), and the 100 bp paired-end sequencing chemistry. All metatranscriptomic data sets were subjected to quality control with FastQC v0.11.3, and adapter removal and quality trimming using BBTools^2^. Thus, three metatranscriptomic data sets were obtained, namely CRF2R7, CRF2R20, and CRF2R68, where R stood for RNA.

All thirteen metagenomic data sets and three metatranscriptomic data sets are accessible at the European Nucleotide Archive of the European Bioinformatics Institute (ENA/EBI) under the study accession number PRJEB38017.

### Taxonomic Analysis of Metagenomic Data

#### Taxonomic Analysis Using Metagenomic Profiling Tools

The quality-trimmed metagenomic sequence reads were used to assess the taxonomic composition of the cocoa fermentation samples using DIAMOND (Buchfink et al., 2015), Kraken 2 (Wood et al., 2019), Kaiju (Menzel et al., 2016), and MetaPhlAn2 (Truong et al., 2015). BLAST (Altschul et al., 1990) was applied for the metagenomic sequence data derived from F2 solely, as the use of this computationally demanding tool for F2 resulted in the detection of only one additional microbial genus compared to the other methods combined, indicating a sufficient degree of genus-level coverage without BLAST.

The BLAST algorithm blastn was used to compare the metagenomic sequence reads to the National Center for Biotechnology Information (NCBI; Bethesda, MD, United States) non-redundant nucleotide database (NCBI-nt; accessed February 2017; NCBI Resource Coordinators, 2016) and a database consisting of bacterial, archaeal, fungal, and viral genomes from RefSeq (accessed February 2017; O’Leary et al., 2016). DIAMOND was used to compare the metagenomic sequence reads to the NCBI non-redundant protein database (NCBI-nr; accessed February 2017; NCBI Resource Coordinators, 2016). The output of BLAST and DIAMOND was parsed with MEGAN 6.7.11 (Huson et al., 2016), using the following parameters: Min Score, 100; Max Expected, 0.01; Min Percent Identity, 0.0; Top Percent, 10.0; Min Support Percent, 0.01; Min Support, 0.00; and LCA Algorithm, Naive. When tabular BLAST/DIAMOND output was used, Min Support was manually set to the equivalent of 0.01% of all reads in the data set.

Kraken 2 was used to compare the metagenomic sequence reads on both the nucleotide level and the protein level to a database composed of bacterial, fungal, archaeal, viral, protozoan, plant, and human complete genome sequences from RefSeq, or amino acid sequences derived thereof. Both databases were constructed using the built-in database creation options of Kraken 2 (accessed August 2018). Kaiju was used to compare the sequence data on the amino acid sequence level to a database constructed by using Kaiju’s built-in database creation options and based on bacterial, archaeal, viral, fungal, and (other) microbial eukaryotic protein sequences from NCBI-nr (accessed July 2018). MetaPhlAn2 was used to estimate the structure of the microbial communities in the samples using the default database of marker gene sequences mpa_v20 (accessed April 2014).

#### Metagenomic Recruitment Plotting

Metagenomic recruitment plotting was performed as described previously (Verce et al., 2019), based on genera represented by more than 0.1% of all reads in any of the 13 metagenomes, according to the results obtained with BLAST, DIAMOND, Kraken 2, or Kaiju, as mentioned above. In the case that several equally good hits were considered as the best hit during filtering with an in-house Python script, a random one was chosen among them to avoid bias, as in such cases the hits are alphabetically listed according to the subject accession number (Shah et al., 2019). The results were adjusted for recent major taxonomic changes regarding the genus *Lactobacillus* (Zheng et al., 2020).

#### Overall Taxonomic Analysis Based on Metagenomic Profiling Tools and Metagenomic Recruitment Plotting

The read-level outputs of several tools were merged to maximize the number of assigned reads, taking the taxonomic changes of the genus *Lactobacillus* into account (Zheng et al., 2020). The outputs were ranked in the following, decreasing order of priority: metagenomic recruitment plotting, Kaiju, Kraken 2 (nucleotide level), Kraken 2 (amino acid level), and DIAMOND. Genus richness, Shannon’s diversity index, and Pielou’s evenness index were calculated with the R package vegan (Oksanen et al., 2019), using the genus-level assignments, whereby only taxa represented by at least 0.1% of all reads were retained, and the categories “Minorities,” “Above genus” and “Unassigned” were retained as pseudo-taxa.

### Co-assembly of Metagenomes, Contig Binning, and Contig Annotation

All 13 metagenomic data sets were co-assembled using the MEGAHIT assembler (Li et al., 2015), with the following parameters: min-count, 1; k-list, 21, 29, 39, 49, 59, 69, 79, 89, 99, 109, 119, 129, 139, 159, and 179; and min-contig-len, 1000. The assembly was imported into anvi’o v5.1 (Eren et al., 2015), wherein a contig database was set up from contigs of at least 2.5 kbp. Contigs longer than approximately 20 kbp were split into parts called splits. An anvi’o profile database was set up by mapping the metagenomic sequence reads from each data set to the co-assembly using Bowtie 2 (Langmead and Salzberg, 2012). Taxonomic information was obtained by classifying the anvi’o-generated gene calls using Kaiju, as mentioned above. Contig/split binning was performed using CONCOCT (Alneberg et al., 2014). The resulting bins were manually refined based on the sequence composition and differential coverage, as well as the completeness and contamination statistics calculated by CheckM (Parks et al., 2015).

The metagenomic bins were annotated with PROKKA (Seemann, 2014). As the primary reference for functional annotation, RefSeq or GenBank protein sequences from selected bacterial species were used, based on the results of the taxonomic analysis, to represent all major bacterial groups present in the metagenomes, namely *A. ghanensis* LMG 23848^*T*^ (GCF_001499675.1), *A. pasteurianus* 386B (GCF_000723785.2), *Acinetobacter populi* PBJ7^*T*^ (GCF_002174125.1), *Cellvibrio japonicus* Ueda 107^*T*^ (GCF_000019225.1), *Dysgonomonas capnocytophagoides* DSM 22835^*T*^ (GCF_000426485.1), *Gluconacetobacter entanii* LTH 4560^*T*^ (GCF_003206495.1), *Komagataeibacter hansenii* ATCC 23769 (GCF_000164395.1), *Liquorilactobacillus cacaonum* DSM 21116^*T*^ (GCF_001436735.1; formerly *Lactobacillus cacaonum*), *Liml. fermentum* IMDO 130101 (GCA_900205745.1), *Liquorilactobacillus nagelii* DSM 13675^*T*^ (GCF_001434225.1; formerly *Lactobacillus nagelii*), *Lacp. plantarum* WCFS1 (GCF_000203855.3), *Paucilactobacillus vaccinostercus* DSM 20634^*T*^ (GCF_001436295.1; formerly *Lactobacillus vaccinostercus*), *Leuconostoc pseudomesenteroides* PS12 (GCF_000686505.1), *Pectobacterium carotovorum* PC1 (GCF_000023605.1), and *Tatumella ptyseos* NCTC 11468^*T*^ (GCF_900478715.1). To facilitate further analyses, orthogroups (OGs) were inferred from predicted protein products from all metagenomic bins using OrthoFinder (Emms and Kelly, 2015). Contigs within the metagenomic bins corresponding to *Hanseniaspora opuntiae* and *S. cerevisiae* were annotated with MAKER2 (Holt and Yandell, 2011), using Saccharomycetales protein sequences from Swiss-Prot (The UniProt Consortium, 2019) as references for functional annotation.

### Metatranscriptomic Analysis

Bacterial, archaeal, and eukaryotic rRNA reads were removed from the metatranscriptomic data sets using SortMeRNA (Kopylova et al., 2012). The resulting sequences were mapped using minimap2 (Li, 2018) to tRNA sequences from the annotated metagenomic bins. Metatranscriptomic sequence reads that did not match any rRNA or tRNA sequences were presumed to be mRNA reads.

Kallisto (Bray et al., 2016) was used to map mRNA reads to coding sequences (CDSs) obtained from the PROKKA or MAKER2 annotations of all metagenomic bins and to estimate expression levels, expressed as transcripts per million (tpm). Due to insufficient quality of the metagenomic bins in the case of AAB, CDSs from the genomes of *A. pasteurianus* 386B (GCF_000723785.2) and *A. ghanensis* LMG 23848^*T*^ (GCF_001499675.1) were used as references for these two major species. The tpm values were initially calculated considering all CDSs in the metagenomic assembly and then re-normalized per metagenomic bin to enable the ranking of CDSs within a metagenomic bin according to their transcription. The CDSs that had less than five reads mapped to them at any time point were excluded. The CDSs were ranked within metagenomic bins according to corrected tpm values and assigned deciles and percentiles, denoting the relative importance of the CDSs within a genome. For instance, the CDS with the highest re-normalized tpm in a given metagenomic bin at a certain time point would be assigned to the 100th percentile and 10th decile.

### Analysis of Genes Related to Pectinolysis

The software tool dbCAN2 (Zhang et al., 2018) was used to identify carbohydrate-active enzymes among the protein sequences obtained from the PROKKA or MAKER2 annotations, or *A. pasteurianus* 386B and *A. ghanensis* LMG 23848^*T*^ assemblies. Only those CDSs were retained that were identified by at least two methods embedded within dbCAN2. After multiple sequence alignment using MUSCLE (Edgar, 2004) and comparison with other sequences using NCBI BLAST, truncated protein sequences were not considered in further analyses. Signal peptides in query protein sequences were identified within dbCAN2 using SignalP 4.0 (Petersen et al., 2011). Due to the presence of simple carbohydrates, the expression of genes related to pectinolysis was assumed to be relatively low in the main species and possibly high in other, less abundant species. Therefore, to increase the detection, CDSs related to pectinolysis with at least one mRNA read mapped were considered as expressed genes. As glycoside hydrolase families GH2, GH3, GH28, GH35, GH43, GH51, GH53, GH78, GH88, GH93, and GH105; polysaccharide lyase families PL1, PL3, PL4, PL9, and PL11; and carbohydrate esterase families CE1, CE8, and CE12 contain enzymes related to pectinolysis (van den Brink and de Vries, 2011), they were further investigated, as well as characterized using NCBI BLAST and the Conserved Domain Database (CDD; Marchler-Bauer et al., 2017) when necessary. Additional information was obtained from CAZypedia^3^, the encyclopedia of carbohydrate-active enzymes.

### Metabolite Target Analysis

#### Preparation of Aqueous Extracts From Cocoa Pulp and Cocoa Beans

The cocoa pulp, together with the testa, was manually separated from the cocoa beans using a scalpel and forceps. Aqueous extracts were prepared from the cocoa pulp and the cocoa beans separately, based on a protocol described for coffee (Zhang et al., 2019). The cocoa pulp or cocoa mass was frozen in liquid nitrogen and ground using a coffee grinder (DeLongi KG49, Treviso, Italy) until a fine powder was obtained. A 0.4 g sample of powdered cocoa pulp or cocoa beans was mixed with 28 mg of EDTA (Merck, Darmstadt, Germany) and 10 mL of an ascorbic acid solution (2.0 mg/mL; Merck). The extractions took place at room temperature for 30 min, using a rotator Stuart SB3 (Cole-Parmer, Stone, Staffordshire, United Kingdom). The mixture was then centrifuged at 6,000 × *g* for 15 min, after which the aliquots of the supernatants were frozen until analysis. All extractions were performed in triplicate and each aqueous extract was analyzed as described below.

#### Quantification of Simple Carbohydrates and Sugar Alcohols

The concentrations of simple carbohydrates (arabinose, fructose, galactose, glucose, and sucrose) and sugar alcohols (arabitol, erythritol, glycerol, mannitol, *myo*-inositol, sorbitol, and xylitol) were determined by high-performance anion exchange chromatography with pulsed amperometric detection (HPAEC-PAD) using an ICS-5000 chromatograph (Dionex, Sunnyvale, CA, United States), as described before (Zhang et al., 2019). Simple carbohydrates were separated using a CarboPac PA-20 column (Dionex). The mobile phase consisted of a mixture of eluents A, B, and C [ultrapure water with 0, 95, and 736 mM NaOH (J.T. Baker, Deventer, The Netherlands), respectively] at a constant flow rate of 0.4 mL/min and the following gradient: 0.0–15.0 min, isocratic 11.0% B; 15.0–15.1 min, linear from 11.0 to 0% B and from 0 to 100% C; 15.1–25.0 min, isocratic 100% C; 25.0–25.1 min, linear from 100 to 0% C and from 0 to 11% B; and 25.1–35.0 min, isocratic 11.0% B. Sugar alcohols were separated using a CarboPac MA-1 column (Dionex). The mobile phase consisted of eluents A and C at a constant flow rate of 0.4 mL/min, according to the following gradient: 0.0–5.0 min, isocratic 6% C; 5.0–31.5 min, linear from 6.0 to 34.0% C; 31.5–31.6 min, linear from 34.0 to 99.0% C; 31.6–50.0 min, isocratic 99.0% C; 50.0–50.1 min, linear from 99.0 to 6.0% C; and 50.1–60.0 min, isocratic 6.0% C. Samples were prepared by adding 100 μL of (diluted) aqueous extract to 900 μL of an internal standard solution consisting of 50% acetonitrile (Sigma-Aldrich) and 10 mg/L of rhamnose (Merck), followed by vortexing for 5 min, microcentrifugation at 20,000 × *g* at room temperature for 15 min, and filtration through a 0.2 μm regenerated cellulose filter (Whatman Uniflo, GE Healthcare Life Sciences, Little Chalfont, Buckinghamshire, United Kingdom).

#### Quantification of Short-Chain Fatty Acids and Low-Molecular-Mass Volatile Organic Compounds

The concentrations of short-chain fatty acids (acetic acid, propionic acid, isobutyric acid, valeric acid, isovaleric acid, and hexanoic acid) and low-molecular-mass volatile organic compounds (acetaldehyde, ethanol, diacetyl, acetoin, ethyl acetate, ethyl lactate, and isoamyl acetate) were determined by gas chromatography with flame ionization detection (GC-FID), using a Focus GC chromatograph, equipped with an FID-80 detector (Thermo Fisher Scientific), and a Stabilwax-DA column (Restek, Bellefonte, PA, United States), as described before (Zhang et al., 2019). Samples of 1 μL were directly injected, applying a split ratio of 1:20. The injector temperature was set to 240°C, the oven temperature increased from the initial 40°C up to 140°C at 10°C/min and then to 230°C at 50°C/min, whereas the detector temperature was set to 250°C. The carrier gas was hydrogen gas, flowing at a rate of 1 mL/min. The make-up gas was nitrogen gas (Praxair, Schoten, Belgium). 1-Butanol was used as an internal standard.

#### Quantification of Organic Acids

The concentrations of citric acid, fumaric acid, gluconic acid, isocitric acid, 5-ketogluconic acid, lactic acid, malic acid, oxalic acid, succinic acid, and quinic acid were determined by ultra-performance liquid chromatography coupled to tandem mass spectrometry (UPLC-MS/MS), using an Acquity UPLC system, equipped with an HSS T3 column and a TQ tandem mass spectrometer with a ZSpray electrospray ionization source (Waters, Milford, MA, United States), as described before (Zhang et al., 2019). The mobile phase consisted of eluents A and B [ultrapure water-methanol mixtures in ratios of 98:2 (v/v) and 5:95 (v/v), respectively, both with 0.2% (v/v) formic acid], according to the following gradient at 0.23 mL/min: 0.0–1.5 min, isocratic 8.0% B; 1.5–3 min, linear from 8.0 to 89.8% B; 3.0–6.0 min, isocratic 89.8% B; 6.0–6.5 min, linear from 89.8 to 8.0% B; and 6.5–10 min, isocratic 8.0% B. Quantification was performed through external calibration in triplicate.

## Results

### Changes of Temperature and pH During Cocoa Fermentation

**FIGURE 1:**
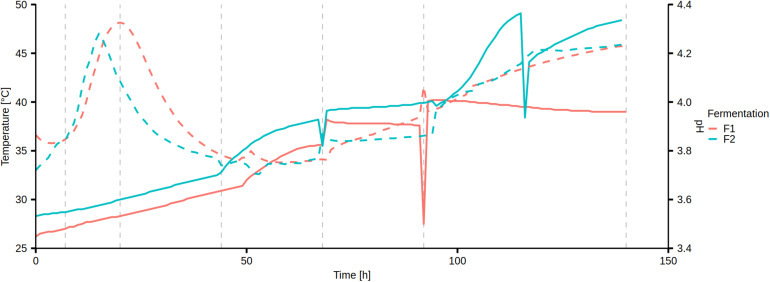
Continuous measurement of temperature (solid curve) and pH (dashed curve) of the Costa Rican cocoa box fermentation processes F1 and F2. The vertical dashed lines represent the sampling time points.

Temperature and pH were measured continuously within the fermenting cocoa pulp-bean mass of the Costa Rican cocoa box fermentation processes F1 and F2 (Figure 1). In fermentation process F1, the temperature increased from an initial 26.2 to 40.2°C after 94 h, after which it stabilized and slowly decreased to a final temperature of 39°C (from 132 to 140 h). In fermentation process F2, the temperature increased from an initial 28.3°C to a maximum of 49.1°C after 115 h, ultimately reaching 48.4°C after 139 h.

The pH changed in a similar fashion in both fermentation processes, starting at pH 3.72–3.87, followed by a temporary increase to pH 4.28–4.33 after 20 h and 15 h in fermentation processes F1 and F2, respectively. After a drop, the pH steadily increased to a final value of 4.23–4.24 in both fermentation processes.

### Taxonomic Analysis of Metagenomic Data

Metagenomic sequencing of 13 samples from three similar Costa Rican cocoa box fermentation processes obtained as a function of time resulted in 13 quality-trimmed data sets with a total size of 11.2 Gbp and with read lengths between 20 and 420 bp (Supplementary Table 1).

**FIGURE 2:**
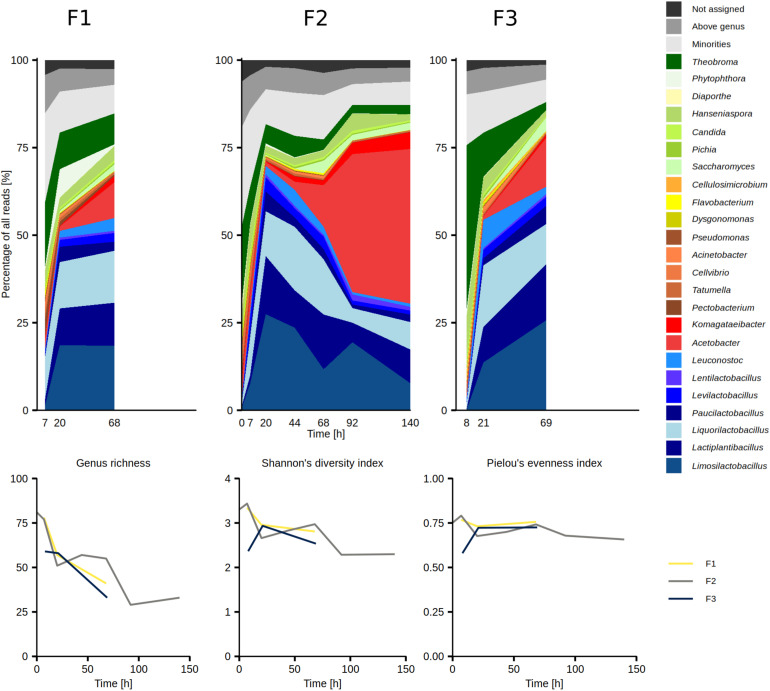
Temporal dynamics of the genus-level diversity based on an overall taxonomic analysis of the metagenomic data sets from the Costa Rican cocoa box fermentation processes F1, F2, and F3 (top row), along with the temporal dynamics of the genus richness, Shannon’s diversity index, and Pielou’s evenness index (bottom row).

The overall taxonomic analysis of the metagenomic data derived from the results of the metagenomic recruitment plotting, Kaiju, Kraken 2 and DIAMOND revealed 23 major microbial genera, arbitrarily defined as genera that were represented by more than 1.0% of all reads in any of the 13 samples (Figure 2 and Table 1). The AAB genus *Acetobacter*, the LAB genera *Lactiplantibacillus, Leuconostoc*, *Limosilactobacillus*, *Liquorilactobacillus*, and *Paucilactobacillus*, the yeast genus *Hanseniaspora*, and the oomycete genus *Phytophthora* reached more than 5.0% of all reads in at least one sample. *Acetobacter* and *Hanseniaspora* were considered as major genera in all 13 metagenomic data sets. Contamination with *Theobroma* DNA reached up to 47.3% of all reads in the metagenomic data set CRF3D8, but it dropped to 12.6% and below by time point 20 h in all three fermentation processes (Figure 2).

**TABLE 1:** Maximum and minimum percentages of reads attributed to microbial genera represented by more than 1.0% of all metagenomic sequence reads in at least one cocoa fermentation sample of the three Costa Rican cocoa box fermentation processes (F1, F2, and F3), according to the overall taxonomic analysis of the metagenomic data and ordered according to the maximum percentage of reads.

Genus	Maximum percentage of reads	Maximum sample	Minimum percentage of reads	Minimum sample
*Acetobacter*	44.18	CRF2D140	1.11	CRF3D21
*Limosilactobacillus*	27.45	CRF2D20	0.05	CRF3D8
*Liquorilactobacillus*	18.15	CRF2D44	0.95	CRF2D0
*Lactiplantibacillus*	16.59	CRF2D20	0.14	CRF3D8
*Hanseniaspora*	12.36	CRF3D8	1.51	CRF3D69
*Phytophthora*	8.10	CRF1D20	0.04	CRF3D69
*Leuconostoc*	8.06	CRF3D21	0.52	CRF2D0
*Paucilactobacillus*	5.64	CRF2D20	0.07	CRF3D8
*Pectobacterium*	4.97	CRF1D7	0.08	CRF3D21
*Komagataeibacter*	4.79	CRF2D140	0.13	CRF3D21
*Levilactobacillus*	4.31	CRF2D7	0.15	CRF2D0
*Saccharomyces*	3.88	CRF3D69	0.03	CRF2D0
*Cellvibrio*	2.91	CRF2D0	0.01	CRF2D92
*Tatumella*	2.77	CRF2D7	0.22	CRF2D140
*Cellulosimicrobium*	2.67	CRF3D8	0.00	CRF2D92
*Candida*	2.11	CRF3D8	0.31	CRF3D69
*Diaporthe*	2.08	CRF2D0	0.01	CRF2D92
*Lentilactobacillus*	1.72	CRF2D92	0.01	CRF3D8
*Acinetobacter*	1.48	CRF3D21	0.07	CRF2D92
*Pseudomonas*	1.28	CRF2D0	0.07	CRF3D69
*Pichia*	1.15	CRF2D7	0.15	CRF3D69
*Flavobacterium*	1.09	CRF2D0	0.04	CRF2D92
*Dysgonomonas*	1.02	CRF2D0	0.03	CRF2D92

Genus richness, diversity, and evenness were the highest in the beginning of the fermentation processes (0–7 h) and exhibited an overall decreasing trend (Figure 2). Of the 97 genera represented by at least 0.1% of all reads in at least one data set, 69 exhibited a generally decreasing trend from the beginning of the fermentation processes, of which 22 and 4 exhibited a transient increase in the middle and at the end of the fermentation processes, respectively (Supplementary Figure 1). Twenty-four microbial genera reached the highest percentages in the middle of the fermentation processes (20–92 h; Supplementary Figure 1) and they comprised 22 LAB genera, the yeast genus *Saccharomyces*, as well as the bacterial genus *Empedobacter*. The most prominent among them were *Limosilactobacillus*, *Liquorilactobacillus*, and *Lactiplantibacillus*, which were represented by up to 27.4, 18.2, and 16.6% of all reads between time points 20 and 44 h, respectively. Viral DNA, mostly from families *Siphoviridae* and *Myoviridae*, also followed this trend. In contrast, four microbial genera exhibited an increasing trend toward the end of the fermentation processes (92–140 h), namely *Acetobacter*, *Komagataeibacter*, *Gluconacetobacter*, and *Zymomonas*. At time points 92 and 140 h, *Acetobacter* represented the largest fraction of all reads, with 39.4 and 44.2%, respectively.

**FIGURE 3:**
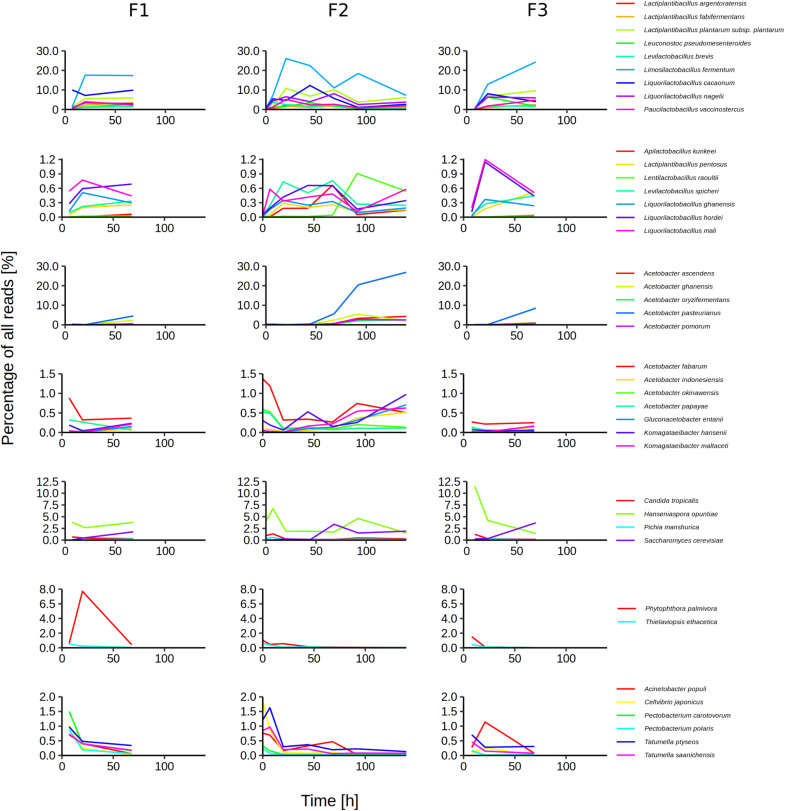
Temporal dynamics of the microbial species during Costa Rican cocoa box fermentation processes (F1, F2, and F3), based on an overall taxonomic analysis of the 13 metagenomic data sets. As major species were considered those that were assigned at least 0.5% of all reads in at least one sample for at least one time point.

On the species level, 119 species or their close relatives were found in one or more of the metagenomic data sets (Supplementary Figure 2 and Supplementary Table 2). Of these species, 40 were regarded as major species, arbitrarily defined as those represented by at least 0.5% of all reads in a data set (Figure 3). Sixteen major species belonged to 8 LAB genera, 12 to the AAB genera *Acetobacter*, *Gluconacetobacter*, and *Komagataeibacter*, 4 belonged to the yeast genera *Candida*, *Hanseniaspora*, and *Saccharomyces*, whereas 6 belonged to the γ-Proteobacterial genera *Acinetobacter*, *Cellvibrio*, *Pectobacterium*, and *Tatumella.* Two species of plant pathogens were also considered to be major species, namely *Phytophthora palmivora* and *Thielaviopsis ethacetica*. The five microbial species that reached the highest percentages of all reads were *A. pasteurianus* (maximum 26.9%), *Liml. fermentum* (maximum 26.0%), *Liql. cacaonum* (maximum 12.3%), *H. opuntiae* (maximum 11.5%), and *Lacp. plantarum* (maximum 10.85%).

Minor bacterial species were for instance *A. senegalensis*, *Acetobacter tropicalis*, *Citrobacter koseri*, *D. capnocytophagoides*, *Enterococcus italicus*, *Frateuria aurantia*, *Fructobacillus pseudoficulneus*, *Gluconobacter japonicus*, *Klebsiella variicola*, *Komagataeibacter saccharivorans*, *Lentilactobacillus farraginis* (formerly *Lactobacillus farraginis*), *Pantoea dispersa*, *Secundilactobacillus collinoides* (formerly *Lactobacillus collinoides*), and *Zymomonas mobilis*. Among the minor yeast species were *Candida ethanolica*, *Hanseniaspora pseudoguilliermondii*, *Pichia occidentalis*, and *Torulaspora delbrueckii*, whereas minor species from other eukaryotic groups were for instance *Phytomonas serpens* and *Moniliophthora roreri*.

### Functional Analysis of Metagenomic and Metatranscriptomic Data

To enable a functional analysis relying on both metagenomic and metatranscriptomic data obtained, the metagenomic sequence reads first had to be assembled into contigs. The resulting 303.5 Mbp metagenomic co-assembly consisted of 155,723 contigs of at least 1,000 bp. After filtering out contigs shorter than 2.5 kbp, the resulting 116.2 Mbp co-assembly consisted of 25,020 contigs with lengths of up to 86.8 kbp. The co-assembly was used for binning and bin refinement, ultimately producing 153 metagenomic bins. Of these bins, five had a completion higher than 90% (Table 2) and four had a redundancy lower than 5%, all belonging to the LAB.

**TABLE 2:** Basic statistics of the bacterial and fungal metagenomic bins with the highest completion or the highest proportion of metatranscriptomic sequence reads mapped from any metatranscriptomic data set of the Costa Rican cocoa box fermentation process F2. All other metagenomics bins were less than 90% complete, and fewer than 10,000 metatranscriptomic reads were mapped to them at any time point.

Metagenomic bin	Size [Mbp]	Completion [%]	Redundancy [%]	Maximum metatranscriptomic reads mapped [%]
*Limosilactobacillus fermentum*	1.63	97.12	1.44	79.30
*Liquorilactobacillus cacaonum*	1.67	95.68	0.72	1.30
*Leuconostoc* sp.	1.52	92.81	0.72	3.68
*Paucilactobacillus vaccinostercus*	2.10	92.09	4.32	3.53
*Liquorilactobacillus nagelii*	1.66	91.37	6.47	2.50
*Lactiplantibacillus plantarum*	2.44	80.58	5.76	7.37
*Saccharomyces cerevisiae*	10.28	77.11	10.84	8.00
*Hanseniaspora opuntiae*	6.53	57.83	2.41	33.0
*Acetobacter pasteurianus*	0.99	25.18	0.00	12.6
*Acetobacter ghanensis*	1.01	21.58	1.44	3.40

After filtering out the rRNA and tRNA sequence reads, the metatranscriptomic data sets consisted of 1,084,654 reads, 1,647,960 reads, and 1,343,560 reads for data sets CRF2R7, CRF2R20, and CRF2R68, respectively. After mapping the metatranscriptomic sequence reads to the CDSs from the metagenomic bins, the top five bins according to the number of mapped reads were those representing *Liml. fermentum*, *H. opuntiae*, *A. pasteurianus*, *S. cerevisiae*, and *Lacp. plantarum* (Table 2). The general functional analysis below will focus mainly on these species.

#### LAB Metabolism

At 7 h of fermentation, 44,230 mRNA reads were mapped to the *Liml. fermentum* metagenomic bin, rising to a maximum of 670,260 mRNA reads at time point 20 h, and dropping to 165,574 mRNA reads by 68 h. The mRNA reads mapped to the *Lacp. plantarum* metagenomic bin followed a similar pattern, namely 21,632 at time point 7 h, 48,284 at time point 20 h, and 22,220 at time point 68 h.

**FIGURE 4:**
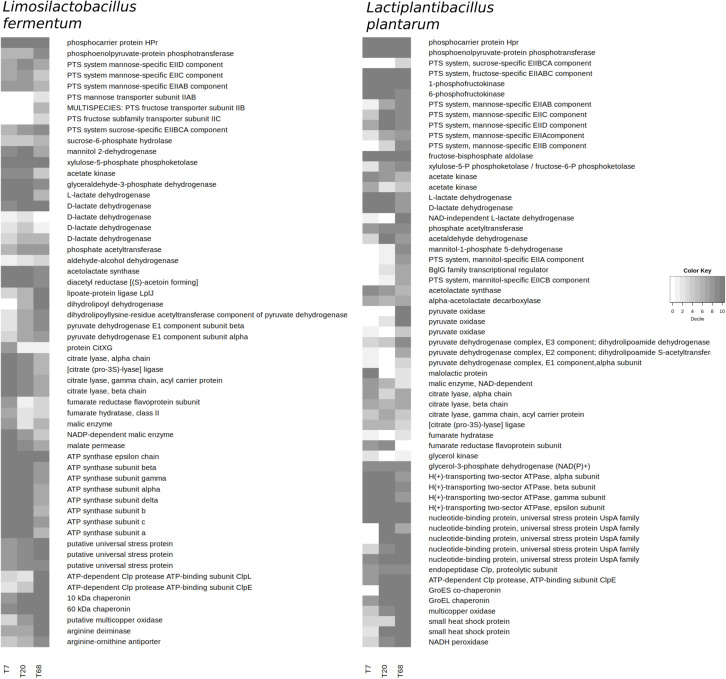
Expression of selected genes at 7, 20, and 68 h of fermentation during the Costa Rican cocoa box fermentation process F2 by *Limosilactobacillus fermentum* and *Lactiplantibacillus plantarum*, according to the mapping of the metatranscriptomic sequence reads to the coding sequences from the metagenomic bins. Shading represents the decile of a coding sequence after ranking according to the expression in a microbial species, with number 10 representing the top 10% of the expressed genes.

The simple carbohydrates typical of cocoa pulp are glucose and fructose, and also sucrose, in the case of unripe cocoa pods. Based on gene expression, both *Liml. fermentum* and *Lacp. plantarum* could import glucose, and possibly fructose *via* a mannose-specific PTS; *Lacp. plantarum* could also import fructose through a fructose-specific PTS component (Figure 4). In *Liml. fermentum*, the expression of fructose-specific PTS components was not found at time point 7 h. In contrast, *Liml. fermentum* could import sucrose through a sucrose-specific PTS and metabolize it, but *Lacp. plantarum* could not. The expression of a mannitol 2-dehydrogenase gene in *Liml. fermentum* throughout the fermentation process enabled the conversion of fructose into mannitol. In contrast, *Lacp. plantarum* could be importing and metabolizing mannitol as a cluster of genes that encode mannitol-specific PTS components and a mannitol-1-phosphate 5-dehydrogenase was expressed.

The gene encoding a xylulose-5-phosphate phosphoketolase, the key enzyme of the heterofermentative pathway, was highly expressed in *Liml. fermentum* (Figure 4). In *Lacp. plantarum*, this gene was expressed to a much lower degree than a gene encoding a fructose-1,6-bisphosphate aldolase, the key enzyme of the homofermentative pathway. A glyceraldehyde-3-phosphate dehydrogenase gene was not found in the *Lacp. plantarum* metagenomic bin, due to a relatively low metagenomic bin completeness (Table 2). In both LAB species, acetyl-P, an important intermediate of the heterofermentative pathway, could be converted into acetate or acetyl-CoA by an acetate kinase and a phosphate acetyltransferase, respectively. Acetyl-CoA could be converted further into ethanol in *Liml. fermentum* because of the expression of a gene encoding a bifunctional aldehyde-alcohol dehydrogenase. Such a gene was not found in the *Lacp. plantarum* metagenomic bin.

Pyruvate, a key branching point in the carbohydrate metabolism, could be converted into L-lactate or D-lactate, in both LAB species, depending on the time point, due to the expression of lactate dehydrogenase genes (Figure 4). Furthermore, *Lacp. plantarum* could oxidize L-lactate into pyruvate using molecular oxygen as an electron acceptor, especially at time point 68 h, due to the expression of a lactate oxidase gene. The expression of an adjacent pyruvate oxidase gene rose from the bottom decile to the top decile of expressed genes, enabling the oxidative decarboxylation of pyruvate into acetyl-P.

Pyruvate could also be converted into α-acetolactate and further into acetoin in *Lacp. plantarum* thanks to the expression of acetolactate synthase and α-acetolactate decarboxylase genes (Figure 4). Despite the absence of the latter in the metagenomic bin, *Liml. fermentum* would be able to reduce diacetyl, spontaneously formed from α-acetolactate, into acetoin and further into 2,3-butanediol. Alternatively, *Liml. fermentum* could convert pyruvate into acetyl-CoA because of highly expressed pyruvate dehydrogenase component genes at time point 68 h. The number of reads mapped to these genes in *Lacp. plantarum* was under the cut-off value.

Citrate could be metabolized by *Liml. fermentum* by cleavage into acetate and oxaloacetate, and further converted into pyruvate, as the genes necessary were highly expressed at 7 h (Figure 4). Their expression dropped in rank over time. The expression of genes encoding some of the citrate lyase components was too low in the *Lacp. plantarum* metagenomic bin to be considered, and the expression levels of the components present showed a less clear trend than for *Liml. fermentum*. Malate could be converted into fumarate and further into succinate, particularly in *Liml. fermentum*.

Genes encoding F-ATPase components were highly expressed in *Liml. fermentum* at time points 7 h and 20 h, followed by a drop at 68 h (Figure 4). Although only four of the seven subunits were found in the *Lacp. plantarum* metagenomic bin, they followed a similar trend. In *Lacp. plantarum*, five genes encoding universal stress proteins were prominently transcribed, two of which throughout the fermentation. The expression of genes encoding universal stress proteins behaved differently in *Liml. fermentum*. An endopeptidase Clp gene was highly expressed in *Lacp. plantarum* throughout the fermentation process, whereas a gene encoding an ATP-binding subunit ClpE was highly expressed at time points 20 and 68 h. Although the former gene was not found in *Liml. fermentum*, the expression of genes encoding the ATP-binding subunits ClpL and ClpE increased sharply by time point 68 h. Genes encoding the chaperonins GroES and GroEL and a multicopper oxidase were highly expressed in both LAB species. Genes encoding an arginine deiminase and an arginine-ornithine antiporter were highly expressed in *Liml. fermentum* at time point 68 h. Additionally, the expression of genes encoding two small heat-shock proteins and an NADH peroxidase showed an increasing trend in *Lacp. plantarum*.

#### Yeast Metabolism

The highest number of mRNA reads was mapped to the *S. cerevisiae* metagenomic bin at time point 7 h (17,872 mRNA reads), which dropped to 13,373 mRNA reads by 68 h. For *H. opuntiae*, the number of mRNA reads dropped from 90,261 at time point 7 h to 2,765 at time point 68 h.

**FIGURE 5:**
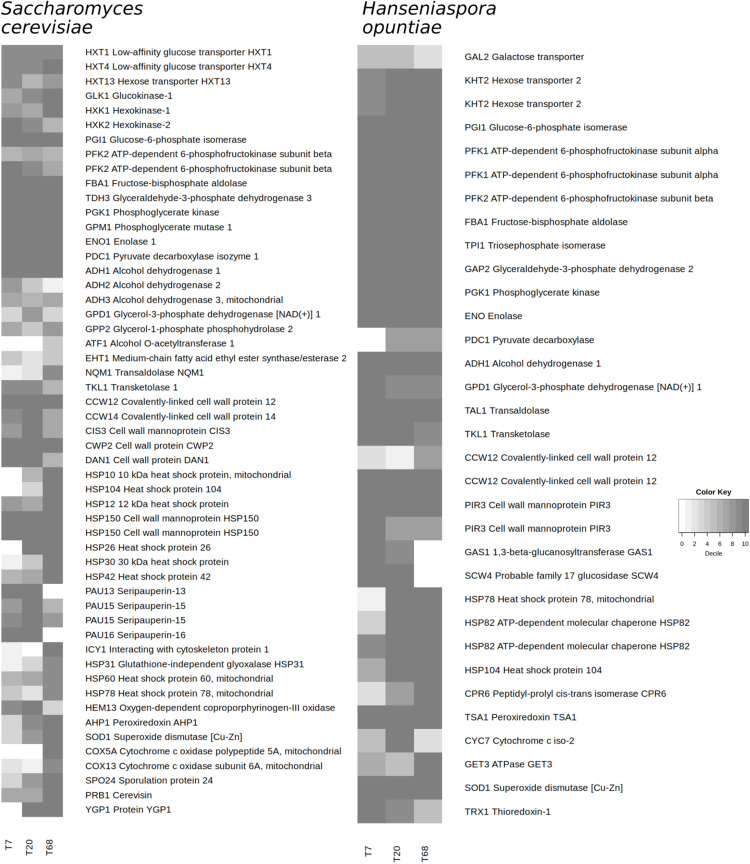
Expression of selected genes at 7, 20, and 68 h of fermentation during the Costa Rican cocoa box fermentation process F2 by *Saccharomyces cerevisiae* and *Hanseniaspora opuntiae*, according to the mapping of the metatranscriptomic sequence reads to the coding sequences from the metagenomic bins. Shading represents the decile of a coding sequence after ranking according to the expression in a microbial species, with number 10 representing the top 10% of the expressed genes.

Expression of invertase genes was not detected for *S. cerevisiae*, except for four reads mapped at 68 h. For both yeast species, multiple genes encoding hexose transporters were expressed at different time points (Figure 5). In *H. opuntiae*, two genes likely encoding hexose transporters were highly expressed. In *S. cerevisiae*, the transcription of genes encoding hexokinases/glucokinases exhibited contrasting trends. No such genes were found in the *H. opuntiae* metagenomic bin.

Even though some genes involved in ethanol fermentation were not found in either metagenomic bin, all other genes involved were in the top decile (Figure 5), demonstrating the importance of this pathway for both yeast species. Besides the decarboxylation of pyruvate into acetaldehyde, pyruvate decarboxylase enables the formation of one acetoin molecule from two acetaldehyde molecules. Acetoin could be reduced into 2,3-butanediol due to an (R,R)-butanediol dehydrogenase that was in the top decile in *S. cerevisiae* at time point 68 h. Genes encoding an alcohol O-acyltransferase and a medium-chain fatty acid ethyl ester synthase/esterase were expressed at mediocre levels, the former only at time point 68 h (Figure 5).

In *S. cerevisiae*, several active genes were related to the environmental conditions, their changes, and the resulting stress. Genes encoding the cell wall proteins CCW12, CWP2, HSP150, and SED1 were highly expressed throughout the fermentation process, whereas the expression of genes encoding the cell wall proteins CCW14, CIS3, DAN1, TIR1, TIR2, and TIR4, the seripauperins PAU13 and PAU15, the cell wall mannoproteins PIR3 and PST1, the heat-shock protein HSP26, and the protein YRO2 with a putative role in acid stress all changed over time (Figure 5). Genes encoding several other heat-shock proteins, the glutathione-independent glyoxalase HSP31, profilin PFY1, and the cell wall protein SPI1 were only highly expressed at time point 68 h. Changes in oxygen availability during the cocoa fermentation process were reflected in the gene transcription related to oxygen-sensing and stress. The expression of the gene encoding the oxygen-dependent coproporphyrinogen-III oxidase HEM13 dropped sharply between time points 20 and 68 h. In contrast, the genes encoding a cytochrome c oxidase polypeptide 5A and subunit 6A, a superoxide dismutase, and peroxiredoxin AHP1 were highly expressed at time point 68 h, but not at time point 7 h. The expression of genes encoding the protein YPG1, cerevisin PRB1, and the sporulation protein SPO24 showed an increasing trend.

In *H. opuntiae*, genes encoding the cell wall (manno)proteins CCW12 and PIR3, as well as the peroxiredoxin TSA1, were highly expressed at all time points (Figure 5). In contrast, the expression of the gene encoding the 1,3-beta-glucanosyltransferase GAS1 decreased from the top to the bottom decile as the fermentation progressed. The expression of a gene encoding the cytochrome c iso-2 was high at time point 20 h, but undetected at 68 h. The genes encoding the heat-shock proteins HSP78 and HSP104, the ATPase GET3, and the peptidyl-prolyl *cis-trans* isomerase CPR6 were highly expressed at time points 20 and 68 h.

#### AAB Metabolism

The number of CDSs from *A. pasteurianus* and *A. ghanensis* genomes, to which at least 5 mRNA reads were mapped, was the highest at time point 68 h (2,250 out of 2816 and 1,373 out of 2610 CDSs, respectively), whereas these numbers were much lower at 7 and 20 h (maximally 157 and 356 CDSs, respectively). Due to this difference, the focus will be on time point 68 h.

**FIGURE 6:**
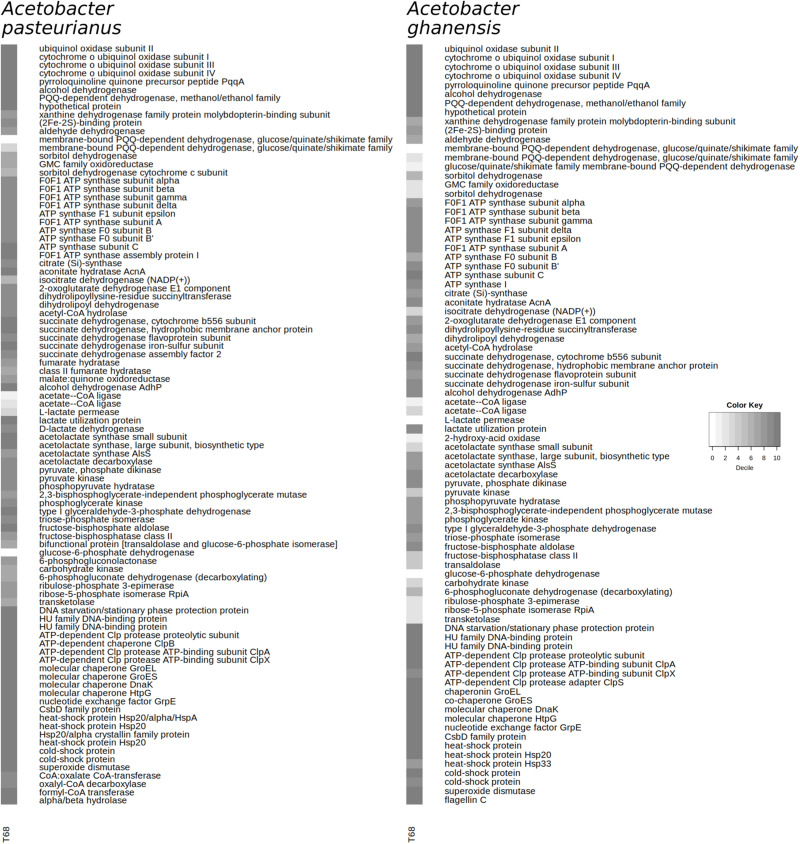
Expression of selected genes at 68 h of fermentation during the Costa Rican cocoa box fermentation process F2 by *Acetobacter pasteurianus* and *Acetobacter ghanensis*, according to the mapping of the metatranscriptomic sequence reads to the coding sequences from the reference genomes. Shading represents the decile of a coding sequence after ranking according to the expression in a microorganism, with number 10 representing the top 10% of the expressed genes.

High expression of pyrroloquinoline quinone (PQQ)- and cytochrome o ubiquinol oxidase-related genes, as well as genes encoding the subunits of a PQQ-dependent alcohol dehydrogenase, emphasized the importance of the respiratory metabolism of ethanol for both AAB species (Figure 6). The genes encoding the three subunits of a PQQ-dependent aldehyde dehydrogenase were expressed to a slightly lower degree (Figure 6). The expression of genes encoding the membrane-bound glucose dehydrogenase and the membrane-bound sorbitol dehydrogenase subunits was even lower in both AAB species. Lastly, most of the genes encoding the ATP synthase subunits were in the top two deciles in both AAB species.

The gene expression related to the tricarboxylic acid cycle in both AAB species ranged mainly between the 9th and the 10th decile (Figure 6). The expression of 16 genes encoding the NADH-quinone oxidoreductase subunits ranged between the 2nd and the 7th decile in *A. pasteurianus*, whereas the expression of the eight subunits with a sufficient number of mapped reads in *A. ghanensis* was spread between the 1st and the 8th decile.

Ethanol assimilation was made possible by the expression of NAD-dependent alcohol dehydrogenase genes in the top 2 deciles in both AAB species (Figure 6). However, the acetate—CoA ligase genes were in the bottom third of all expressed genes in both AAB species.

The expression of lactate utilization-enabling genes was found in both AAB species (Figure 6). Although the expression of an L-lactate permease gene was low, the genes encoding a D-lactate dehydrogenase and a lactate utilization protein were highly expressed in both AAB species. The conversion of lactate into pyruvate would provide input for gluconeogenesis, as well as acetolactate and acetoin formation. The genes encoding the acetolactate synthase subunits were in the top decile in *A. pasteurianus* (Figure 6). Another acetolactate synthase gene was in the 8th decile, whereas an acetolactate decarboxylase gene directly upstream was in the 9th decile in both AAB species.

The gluconeogenesis-related genes were expressed in the range between the 8th and the 10th decile in *A. pasteurianus* and between the 5th and the 9th decile in *A. ghanensis* (Figure 6). In both AAB species, the expression of the bifunctional transaldolase/glucose-6-phosphate isomerase gene was lower than the expression of other gluconeogenesis-related genes. The pentose phosphate pathway-related genes were expressed in the range between the 7th and the 8th decile in *A. pasteurianus*, except for the glucose-6-phosphate dehydrogenase gene, whose expression was very low. In contrast, in *A. ghanensis*, all pentose phosphate pathway-related genes were in the bottom half of the expressed genes, and the number of mRNA reads mapped to the 6-phosphogluconolactonase gene being under the cut-off value.

The expression of genes encoding the Dps protein, which is involved in DNA protection during starvation, two HU family DNA-binding proteins, the ATP-dependent Clp protease proteolytic subunit, the subunit ClpB, and the ATP-binding subunits ClpA and ClpX, the molecular chaperones GroEL, GroES, DnaK, and HtpG, the nucleotide exchange factor GrpE, and a CsbD family protein was in the top decile in both AAB species (Figure 6). In *A. ghanensis*, expression of the ATP-dependent Clp protease adapter ClpS gene was also in the top decile. In *A. pasteurianus*, the expression of genes encoding four Hsp20 family heat-shock proteins and two cold-shock proteins was in the top decile, whereas in *A. ghanensis*, the expression of genes encoding two heat-shock proteins and one cold-shock protein was in the top decile. A gene encoding a superoxide dismutase was among the top 1 and 3% of all expressed genes in *A. pasteurianus* and *A. ghanensis*, respectively.

Genes necessary for oxalate detoxification were found in *A. pasteurianus* solely and were expressed between the 9th and the 10th decile. Expression of an esterase-related gene was also in the top decile. In contrast, motility genes were found in *A. ghanensis* solely and the gene encoding flagellin was among its top 2% of expressed genes. However, expression of other genes involved in motility and chemotaxis was spread across the whole range from the 4th to the 89th percentile.

#### Analysis of Genes Related to Pectinolysis

**FIGURE 7:**
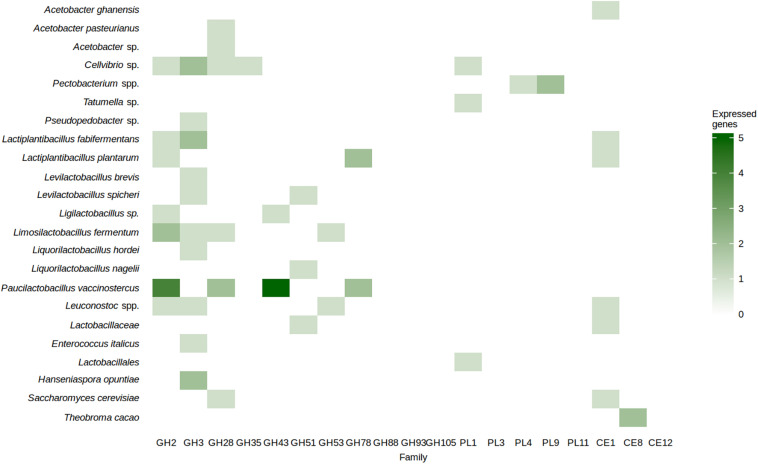
Number of coding sequences encoding carbohydrate-active enzymes from families related to pectin degradation per metagenomic bin derived from the 13 metagenomes of the Costa Rican cocoa box fermentation processes examined, expressed as a heatmap.

In total, 61 CDSs that encoded carbohydrate-active enzymes from protein families relevant to pectinolysis were expressed (Figure 7). They were associated with AAB species, *Cellvibrio* sp., the enterobacterial genera *Pectobacterium* and *Tatumella*, LAB species, *H. opuntiae*, *S. cerevisiae*, and *T. cacao* itself. The families that contain enzymes involved in the cleavage of the main chain of pectins, namely GH28, PL1, PL3, PL4, PL9, and PL11, were represented in *A. pasteurianus*, *Acetobacter* sp., *Cellvibrio* sp., *Pectobacterium* sp., *Liml. fermentum*, *Paul. vaccinostercus*, an unidentified LAB species, and *S. cerevisiae* (Figure 7). Signal peptides were found in GH28 family proteins encoded by *Cellvibrio* sp. and *Acetobacter* sp., PL1 family proteins encoded by an unidentified LAB species and a *Tatumella* sp., and PL4 and PL9 family proteins encoded by *Pectobacterium* spp. The protein family GH43, containing enzymes involved in the degradation of the arabinan side chains of pectins, was represented in *Paul. vaccinostercus* and a *Ligilactobacillus* species. The protein family GH53, containing enzymes involved in the degradation of galactan side chains of pectins, was represented in *Liml. fermentum* and *Leuconostoc* spp. A pectin methyl esterase was expressed by *T. cacao.*

### Metabolite Target Analysis

#### Course of Simple Carbohydrates and Sugar Alcohols

**FIGURE 8:**
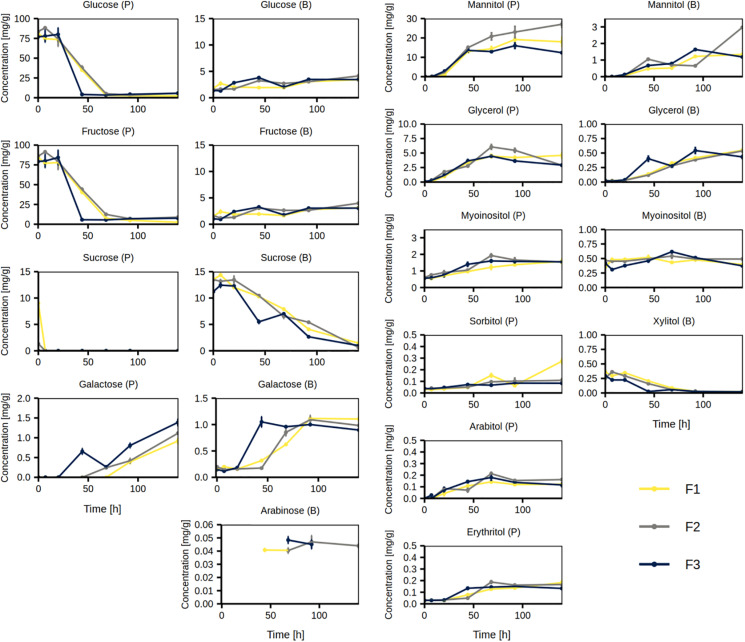
Concentrations of simple carbohydrates and sugar alcohols measured in the cocoa pulp (P) and cocoa beans (B) during the Costa Rican cocoa box fermentation processes F1, F2, and F3. The concentrations were determined in triplicate. The error bars represent standard deviations.

The most abundant simple carbohydrates in the cocoa pulp in the beginning of the Costa Rican cocoa box fermentation processes were fructose (ca. 83 mg/g) and glucose (ca. 80 mg/g), which were almost depleted by 44 h (F3)–68 h (F1 and F2; Figure 8). Low concentrations of sucrose (up to ca. 10 mg/g, F1) in the cocoa pulp were depleted already by 7 h. In the cocoa beans, the concentrations of fructose and glucose slowly increased over time, whereas the concentrations of sucrose decreased over time during all three fermentation processes. The concentrations of galactose in the cocoa pulp increased until the end of the fermentation processes, however, it only increased until the middle of the fermentation processes in the cocoa beans. Arabinose was not quantifiable in the cocoa pulp, but was quantifiable at some time points in the cocoa beans and it followed an increasing trend with a small drop at the end of the fermentation processes (Figure 8).

The concentrations of mannitol increased throughout the three fermentation processes in both cocoa pulp and cocoa beans (Figure 8). In the cocoa pulp, it reached the highest concentration at 92 h during fermentation processes F1 and F3 (ca. 16–19 mg/g) and at 140 h in fermentation process F2 (ca. 29 mg/g). The concentrations of glycerol in the cocoa pulp increased until the middle of the fermentation processes, followed by a decrease. In contrast, the concentrations of glycerol in the cocoa beans steadily increased. The concentrations of *myo*-inositol in the cocoa pulp and cocoa beans increased only slightly or remained relatively stable, respectively. The concentrations of sorbitol, arabitol, and erythritol slightly increased in the cocoa pulp, whereas in the cocoa beans, they were under the limit of quantification. In contrast, xylitol could only be quantified in the cocoa beans, where it was depleted by the end of the fermentation processes.

#### Course of Short-Chain Fatty Acids and Low-Molecular-Mass Volatile Organic Compounds

**FIGURE 9:**
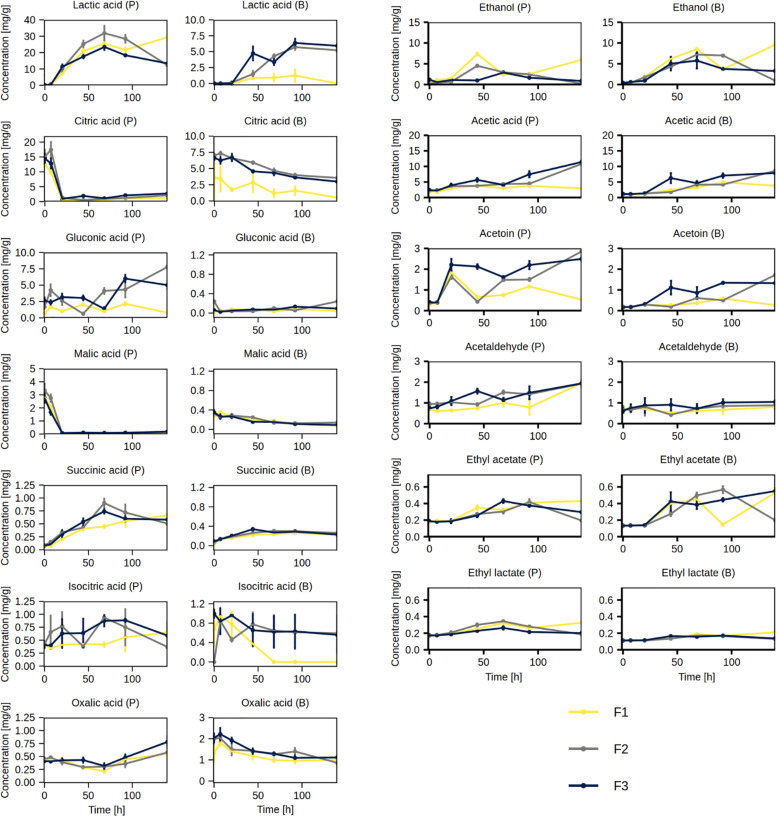
Concentrations of organic acids, short-chain fatty acids, and volatile organic compounds measured in the cocoa pulp (P) and cocoa beans (B) during the Costa Rican cocoa box fermentation processes F1, F2, and F3. The concentrations were determined in triplicate. The error bars represent standard deviations.

The main volatile organic compounds produced during the cocoa fermentation processes were ethanol and acetic acid (Figure 9). Both compounds were present in the cocoa pulp and cocoa beans. The concentrations of ethanol in the cocoa pulp peaked at 44 h (F1 and F2) or 68 h (F3), generally followed by a decrease, and a similar trend was found in the cocoa beans, albeit with a delay. The concentrations of acetic acid in the cocoa pulp increased in fermentation processes F2 and F3, reaching the highest concentration at 140 h, but not in F1. Similarly, the concentrations of acetic acid in the cocoa beans increased in fermentation processes F2 and F3 more than in F1.

The concentrations of acetoin in the cocoa pulp increased sharply in all three fermentation processes between 7 and 20 h. This was followed by a decrease in fermentation process F1, a transient decrease in F2, and a generally stable concentration in F3. In the cocoa beans, the concentrations of acetoin generally increased by the end of the fermentation processes, although the increase was low for F1. The concentrations of acetaldehyde in the cocoa pulp increased by the end of the fermentation processes; however, its concentrations remained stable throughout the fermentation processes in the cocoa beans. Ethyl acetate and ethyl lactate were also produced in all fermentation processes. Their concentrations increased in both cocoa pulp and cocoa beans, although the concentrations of ethyl acetate increased more than those of ethyl lactate, especially in the cocoa beans (Figure 9). The concentrations of propionic acid, isobutyric acid, isovaleric acid, valeric acid, hexanoic acid, and isoamyl acetate were between 0.4 and 1.0 mg/g in both the cocoa pulp and cocoa beans and remained stable throughout the fermentation processes (Supplementary Figure 3).

#### Course of Organic Acids

Initially, the most abundant organic acids in the cocoa pulp were citric acid (ca. 15 mg/g) and malic acid (ca. 3 mg/g), although both were depleted by 20 h of fermentation (Figure 9). The most abundant organic acid produced during the fermentation processes was lactic acid, which peaked by 68 h (ca. 20–30 mg/g), followed by a decrease. In the cocoa beans, the decrease in citric acid and malic acid concentrations, as well as the increase in lactic acid concentrations, were slower. The gluconic acid and succinic acid concentrations exhibited an increasing trend in the cocoa pulp. The former were barely quantifiable in the cocoa beans and the latter increased slightly. The concentrations of isocitric acid and oxalic acid decreased in the cocoa beans. However, the concentrations of oxalic acid increased in the cocoa pulp, whereas the trend was variable for isocitric acid.

## Discussion

Up to now, cocoa fermentation processes have been studied using culture-dependent methods, as well as PCR-based culture-independent methods, with few exceptions (Illeghems et al., 2012; Agyirifo et al., 2019; Lima et al., 2020), revealing key players for these fermentation processes, as well as major metabolic activities. Besides a significant expansion of cocoa fermentation shotgun metagenomic data available, the present study of Costa Rican cocoa box fermentation processes introduced metatranscriptomics into cocoa fermentation research, providing a deeper characterization of metabolic activities of previously established key players, as well as an insight into previously overlooked microbial processes and interactions therein.

**FIGURE 10:**
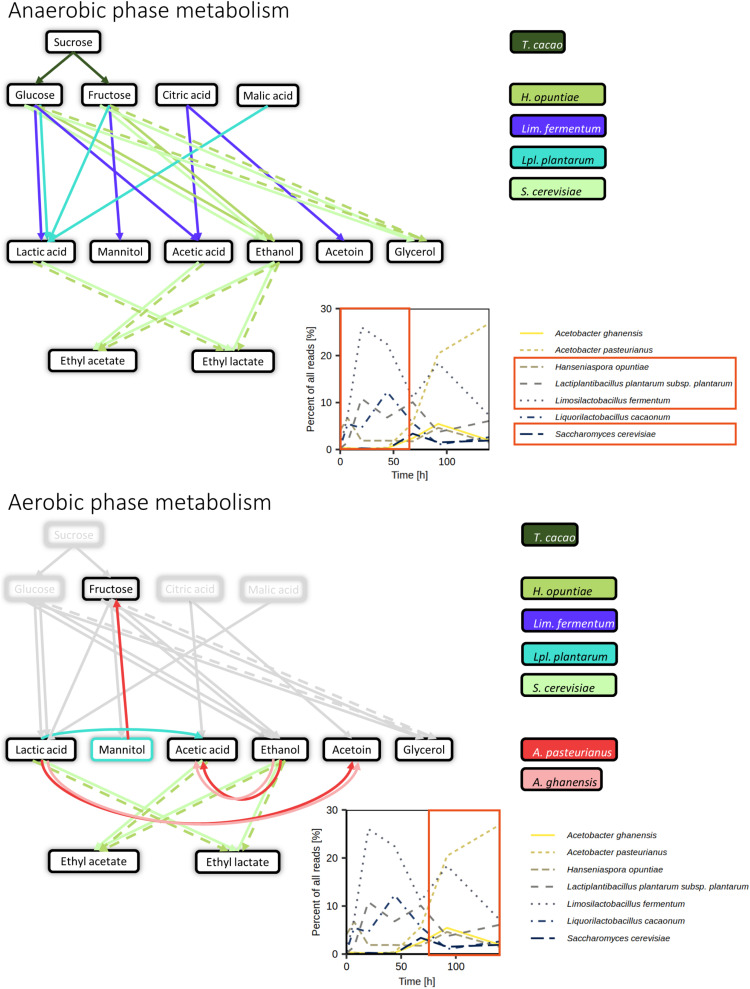
Summary of the anaerobic (above) and aerobic (below) metabolism in the Costa Rican cocoa box fermentation processes based on the metagenomic and metatranscriptomic analyses, as well as metabolite target analysis. Arrow colors indicate the (micro)organisms mainly influencing specific metabolite conversions based on gene expression and representation in metagenomic data. Dashed arrows indicate reactions likely occurring based on previous knowledge but without direct support from the metagenomic and metatranscriptomic data. Percentages of reads attributed to the seven main microbial species were derived from metagenomic data sets of fermentation process F2.

In general, the cocoa fermentation processes performed in the present study exhibited temperature, pH, microbial communities, and metabolite profiles typical for this kind of spontaneous fermentation processes (Figure 10; Schwan and Wheals, 2004; De Vuyst and Weckx, 2016; De Vuyst and Leroy, 2020). Yet, the differences between the fermentation processes examined, especially in the temporal trends of the microbial species and metabolites, underlined the variable nature of spontaneous cocoa fermentation processes. For instance, agricultural practices influence the level of microbial diversity (Papalexandratou et al., 2011b, 2013). Indeed, the practices at the farm where the fermentations were performed were more standardized than on an average farm; however, in contrast to manual cocoa pod opening, the high throughput of the mechanical opening of cocoa pods did not allow occasional pieces of cocoa pod husks to be removed, likely leading to a higher microbial load from the outside of the cocoa pods in the beginning of the fermentation processes. The decrease of the microbial diversity over the course of the three fermentation processes indicated increasing environmental pressures, due to the consumption and depletion of simple carbohydrates, increasing ethanol concentrations, increasing acidic stress caused by the production of lactic acid and acetic acid, and an increasing temperature of the fermenting cocoa pulp-bean mass.

The major microbial species found in the present study, namely *Liml. fermentum*, *Liql*. *cacaonum*, *Lacp. plantarum*, *A. pasteurianus*, *A. ghanensis, H. opuntiae*, and *S. cerevisiae*, have been found in most cocoa fermentation processes before, reaffirming their role as the key players during the fermentation of cocoa pulp-bean mass (De Vuyst and Weckx, 2016; De Vuyst and Leroy, 2020), whereas *Liql. cacaonum* has been found less frequently than the other LAB species since its discovery in 2009 (De Bruyne et al., 2009). This indicated a sufficient coverage of the major microorganisms by culture-dependent methods so far. Moreover, it could be shown that the yeast species *H. opuntiae* was the most prominent key species in the very beginning of the cocoa fermentation processes under investigation, followed by a quick rise of *Liml. fermentum, Lacp. plantarum* and *Liql. cacaonum*, and then persistence of the former two LAB species and the rise of *A. pasteurianus* and *A. ghanensis* until the end of these processes. Although the prevailing yeast species was *H. opuntiae*, *S. cerevisiae* reached a similar level by the middle of the fermentation processes. This confirms the common microbial community dynamics of cocoa fermentation processes, which can be ascribed to the changing environmental conditions (substrate availability and/or competition and metabolites produced) and microbial interactions (such as cross-feeding, for instance between yeasts and AAB; De Vuyst and Weckx, 2016; De Vuyst and Leroy, 2020).

Major genera that have not been reported in cocoa fermentation processes before included *Cellvibrio* and *Dysgonomonas.* Whereas the former genus is typically saprophytic, the latter one is found in many terrestrial environments and is particularly enriched in insect systems (Gardner, 2016; Bridges and Gage, 2020). *Acinetobacter* and *Pectobacterium* have previously been identified in cocoa fermentation processes through shotgun metagenomics or amplicon sequencing (Illeghems et al., 2012; Bortolini et al., 2016; Agyirifo et al., 2019; Serra et al., 2019). Other bacterial genera, such as *Flavobacterium* and *Pseudomonas*, have been isolated and identified phenotypically before (Balogu and Onyeagba, 2017). All these genera were the most prominent in the beginning of the three fermentation processes, when the microbial diversity was the highest, reflecting a wide inoculation event, likely from a plant-related origin. Notably, the AAB genus *Komagataeibacter* has been reported before as different *Gluconobacter* and *Gluconacetobacter* species, as the genus name *Komagataeibacter* was only established in 2012 (Garcia-Armisen et al., 2010; Papalexandratou et al., 2011c; Yamada et al., 2012; Hamdouche et al., 2015; Visintin et al., 2016). Similarly, most LAB species found in this study have recently been reclassified and were thus reported accordingly (Zheng et al., 2020).

The microorganisms belonging to the minor genera found in the present study likely originated from soil and other environmental sources, for instance *Sphingobacterium* spp. and *Streptomyces* spp. (Goodfellow et al., 2010; Kämpfer, 2012). The present study also revealed the presence of plant-associated or endophytic microorganisms in the fermenting cocoa pulp-bean mass, whether or not pathogenic, represented by the genera *Thielaviopsis*, *Phytomonas, Colletotrichum, Moniliophthora, Chryseobacterium*, and *Acholeplasma* (the latter possibly *Phytoplasma*; Mitchell, 2004; Mejía et al., 2008; Kube et al., 2014; Mbenoun et al., 2016). Some of these microorganisms have not been detected in cocoa fermentation processes before, but they likely have more relevance to plant health than to cocoa fermentation. For instance, *Phytophthora* and *Moniliophthora* contain species that cause cocoa tree diseases and they were presumably detected due to small pieces of infected cocoa pods inadvertently being present in the sample during DNA extraction, most likely due to the automated pod opening method applied at the farm, not allowing a full manual removal of pod particles. As their presence in cocoa fermentation would only be expected if some of the cocoa pods came from infected plants, which should generally be prevented by careful pod selection on the plantations, not much is known about their possible impact on the cocoa fermentation process.

The temporal dynamics of the metabolites in the cocoa pulp resulted from the interplay of the different microbial groups involved in the three cocoa fermentation processes. For instance, the rapid depletion of citric acid and the accumulation of mannitol in the cocoa pulp could be linked to the growth and activities of *Liml. fermentum*, as reported for isolates of this LAB species before (Lefeber et al., 2010). Furthermore, the differences in the temporal dynamics of acetoin between the three fermentation processes could be attributed to a varying presence or capability of microorganisms to reduce acetoin, produced by yeasts and LAB, to 2,3-butanediol, before AAB produce more acetoin (Lefeber et al., 2010, 2011; Moens et al., 2014). Apart from lactic acid utilization by AAB, some decrease in the lactic acid concentrations could be attributed to its utilization by *Lacp. plantarum* under aerobic conditions, as described before (Murphy et al., 1985). However, lactate dehydrogenases and not lactate oxidases are thought to enable this activity in *Lacp. plantarum* (Goffin et al., 2004), obscuring the role of the lactate oxidase gene expressed. Lactate utilization is supported by the expression of pyruvate oxidase and an NADH peroxidase (de Angelis and Gobbetti, 2004; Goffin et al., 2004). Oxalic acid is naturally present in cocoa beans and may play a role in the final flavor (Holm et al., 1993; Afoakwa, 2016). The decrease of its concentration in the cocoa beans could be due to its diffusion into the cocoa pulp and subsequent degradation by *A. pasteurianus*. Besides fructose and glucose, other monosaccharides and sugar alcohols, either present in the plant tissues, emerging from the degradation of plant tissues, or produced by the microorganisms present, may act as resources for the persistence of microbial activity. For instance, galactose, which was never measured during cocoa fermentation processes before, was likely made accessible by the degradation of the rhamnogalacturonan components of pectin (Meersman et al., 2017) and/or the hydrolysis of anthocyanins such as cyanidin-3-β-D-galactoside and cyanidin-3-α-arabinoside (De Taeye et al., 2016; De Vuyst and Leroy, 2020). Similarly, mannitol produced by *Liml. fermentum* could partly be consumed by AAB species and *Lacp. plantarum*, resulting in the production of fructose and lactic acid and/or acetic acid, respectively (Chakravorty, 1964; Sedewitz et al., 1984; Zaunmüller et al., 2006; Moens et al., 2014).

The environmental conditions in the fermenting cocoa pulp-bean mass became increasingly stressful, as indicated by the expression of genes related to heat, acid, and oxidative stress responses (Papadimitriou et al., 2016). For instance, temperatures approaching 40°C are already beyond the growth temperature optimum of yeasts such as *S. cerevisiae* and *H. opuntiae*, and can be considered high, relative to the common temperature range for growth of these microorganisms (Lawrence et al., 1959; Vaughan-Martini and Martini, 2011). Furthermore, it has been shown before that heat shock proteins are produced by *S. cerevisiae* at temperatures approaching 40°C, presumably to allow the cells to develop an induced thermotolerance in anticipation of possibly higher temperatures to come, for instance in the case of Hsp104 (Sanchez and Lindquist, 1990). Also, multicopper oxidase genes were expressed in *Liml. fermentum* and *Lacp. plantarum*, contributing to copper stress response, biogenic amine degradation, or possibly as a response to the presence of cocoa polyphenols (Rademacher and Masepohl, 2012; Callejón et al., 2016).

Pectinolysis has previously been attributed to endo-polygalacturonase activity by yeast species (Schwan et al., 1997; Crafack et al., 2013; Samagaci et al., 2016; Meersman et al., 2017) and to pectinolytic bacteria such as *Bacillus* and the enterobacteria *Erwinia*, *Pantoea* and *Tatumella* (Ouattara et al., 2008; Papalexandratou et al., 2013, 2019; Hamdouche et al., 2015; De Vuyst and Leroy, 2020). The present study suggested that other microorganisms may play a role in the degradation of the main pectin chains as well, such as species of *Cellvibrio* and *Pectobacterium*, which are known degraders of plant cell wall materials (Hugouvleux-Cotte-Pattat et al., 2014; Gardner, 2016). It has been hypothesized that *Cellvibrio* species degrade pectins mainly to liberate cellulose from the surrounding pectin (Gardner, 2016). Yet, other microorganisms may play a role in the degradation of pectin side chains as well. For instance, several genes encoding the family GH43 enzymes were attributed to *Paul. vaccinostercus*, a nutritionally fastidious LAB species depending on, for instance, pentoses (Dellaglio et al., 2006). It has been found in the fermentation step of wet coffee processing too (Zhang et al., 2019). This family of enzymes includes α-L-arabinofuranosidases, endo-α-L-arabinanases, and β-xylosidases. Finally, the pectin methyl esterase expressed by *T. cacao* may have made the pectin breakdown easier for the microorganisms, indicating a synergistic action of endogenous and microbial enzymes. However, as plant gene expression was not targeted, expression of other genes potentially involved in pectinolysis could not be investigated. Based on the presence of this diverse set of (micro)organisms with the ability to contribute to cocoa pulp pectinolysis, it could be hypothesized that the pectin degradation process could benefit from dedicated microbial interactions, and indeed, synergistic effects in the pectin degradation process have been indicated before (Ouattara et al., 2020).

## Conclusion

This study dealt with three similar Costa Rican cocoa fermentation processes and revealed the presence of microbial species known to be major players in cocoa pulp-bean mass fermentations, in particular *H. opuntiae*, *Liml. fermentum*, *Lacp. plantarum*, *S. cerevisiae*, *A. pasteurianus*, and *A. ghanensis.* The consumption of major carbohydrates and organic acids was linked to *H. opuntiae*, *S. cerevisiae*, *Liml. fermentum*, and *Lacp. plantarum*, producing mainly ethanol, lactic acid, acetic acid, mannitol, and acetoin. In the later stage of the fermentation processes, ethanol and lactic acid were consumed by AAB, producing acetic acid and acetoin. However, genes enabling previously overlooked metabolic activities were expressed too, for instance regarding lactic acid and mannitol utilization by *Lacp. plantarum*. Although the present study revealed a wide variety of microorganisms in the beginning of the fermentation processes, their diversity decreased over time, which paralleled the increasing stress in the cocoa fermentation environment. Some of the microorganisms present in the beginning of the fermentation processes had a potential role in pectin degradation, emphasizing the complexity of this process and presenting it as a synergistic effort of different microbial communities, and possibly that of the host plant too. The knowledge gained in this study could be beneficial in the search and development of adapted functional starter cultures to steer cocoa fermentation processes.

## Data Availability Statement

The datasets presented in this study can be found in online repositories. The names of the repository/repositories and accession number(s) can be found below: https://www.ebi.ac.uk/ena/browser/view/PRJEB38017.

## Author Contributions

MV performed the sampling, sample processing, bioinformatics, and laboratory analyses. JS contributed to sample processing and bioinformatics. CHA and RM-B coordinated the logistics of the field trials, guided the execution of sample processing, and provided the local knowledge on Costa Rican cocoa fermentations. LD and SW designed and coordinated the study. MV, LD, and SW wrote the manuscript. All authors read and approved the final manuscript.

## Conflict of Interest

The authors declare that the research was conducted in the absence of any commercial or financial relationships that could be construed as a potential conflict of interest.

## Footnotes

1 http://www.bioinformatics.babraham.ac.uk/projects/fastqc/

2 https://sourceforge.net/project/bbmap/

3 www.cazypedia.org

## References

[B1] Afoakwa E. O. (2016). *Chocolate Science and Technology.* United Kingdom: Wiley- Blackwell.

[B2] Agyirifo D. S. Wamalwa M. Plas Otwe E. Galyuon I. Runo S. Takrama J. (2019). Metagenomics analysis of cocoa bean fermentation microbiome identifying species diversity and putative functional capabilities. *Heliyon* 5:e02170. 10.1016/j.heliyon.2019.e02170 31388591 PMC6667825

[B3] Alneberg J. Bjarnason B. S. de Bruijn I. Schirmer M. Quick J. Ijaz U. Z. (2014). Binning metagenomic contigs by coverage and composition. *Nat. Methods* 11 1144–1146. 10.1038/NMETH.3103 25218180

[B4] Altschul S. F. Gish W. Miller W. Myers E. W. Lipman D. J. (1990). Basic local alignment search tool. *J. Mol. Biol.* 215 403–410. 10.1016/S0022-2836(05)80360-2 2231712

[B5] Aranda P. S. LaJoie D. M. Jorcyk C. L. (2012). Bleach gel: a simple agarose gel for analyzing RNA quality. *Electrophoresis* 33 366–369. 10.1002/elps.201100335 22222980 PMC3699176

[B6] Balogu T. V. Onyeagba A. R. (2017). Polyphenol and microbial profile of on-farm cocoa beans fermented with selected microbial consortia. *Appl. Food Biotechnol.* 4 229–240. 10.22037/afb.v4i4.16845 29708228

[B7] Bortolini C. Patrone V. Puglisi E. Morelli L. (2016). Detailed analyses of the bacterial populations in processed cocoa beans of different geographic origin, subject to varied fermentation conditions. *Int. J. Food Microbiol.* 236 98–106. 10.1016/j.ijfoodmicro.2016.07.004 27458718

[B8] Bray N. L. Pimentel H. Melsted P. Pachter L. (2016). Near-optimal probabilistic RNA-seq quantification. *Nat. Biotechnol.* 34 525–527. 10.1038/nbt.3519 27043002

[B9] Bridges C. M. Gage D. J. (2020). *Development and application of aerobic, chemically defined media for Dysgonomonas*. biorXiv, 2020.08.11.247353 [Preprint]. Available online at: https://www.biorxiv.org/content/10.1101/2020.08.11.247353v1 (Accessed November 3, 2020). 10.1016/j.anaerobe.2020.102302 33271360

[B10] Buchfink B. Xie C. Huson D. H. (2015). Fast and sensitive protein alignment using DIAMOND. *Nat. Methods* 12 59–60. 10.1038/nmeth.3176 25402007

[B11] Callejón S. Sendra R. Ferrer S. Pardo I. (2016). Cloning and characterization of a new laccase from *Lactobacillus plantarum* J16 CECT 8944 catalyzing biogenic amines degradation. *Appl. Microbiol. Biotechnol.* 100 3113–3124. 10.1007/s00253-015-7158-0 26590586

[B12] Camu N. De Winter T. Verbrugghe K. Cleenwerck I. Vandamme P. Takrama J. S. (2007). Dynamics and biodiversity of populations of lactic acid bacteria and *acetic acid bacteria* involved in spontaneous heap fermentation of cocoa beans in ghana. *Appl. Environ. Microbiol.* 73 1809–1824. 10.1128/AEM.02189-06 17277227 PMC1828797

[B13] Camu N. González A. De Winter T. Van Schoor A. De Bruyne K. Vandamme P. (2008). Influence of turning and environmental contamination on the dynamics of populations of lactic acid and *acetic acid bacteria* involved in spontaneous cocoa bean heap fermentation in Ghana. *Appl. Environ. Microbiol.* 74 86–98. 10.1128/AEM.01512-07 17993565 PMC2223199

[B14] Chakravorty M. (1964). Metabolism of mannitol and induction of mannitol 1-phosphate dehydrogenase in *Lactobacillus plantarum*. *J. Bacteriol.* 87 1246–1248. 10.1128/JB.87.5.1246-1248.1964 5874545 PMC277181

[B15] Crafack M. Mikkelsen M. B. Saerens S. Knudsen M. Blennow A. Lowor S. (2013). Influencing cocoa flavour using *Pichia kluyveri* and *Kluyveromyces marxianus* in a defined mixed starter culture for cocoa fermentation. *Int. J. Food Microbiol.* 167 103–116. 10.1016/j.ijfoodmicro.2013.06.024 23866910

[B16] Daniel H.-M. Vrancken G. Takrama J. F. Camu N. De Vos P. De Vuyst L. (2009). Yeast diversity of ghanaian cocoa bean heap fermentations. *FEMS Yeast Res.* 9 774–783. 10.1111/j.1567-1364.2009.00520.x 19473277

[B17] de Almeida S. D. F. O. Silva L. R. C. Junior G. C. A. C. Oliveira G. da Silva S. H. M. Vasconcelos S. (2019). Diversity of yeasts during fermentation of cocoa from two sites in the brazilian amazon. *Acta Amazonica* 49 64–70. 10.1590/1809-4392201703712

[B18] de Angelis M. Gobbetti M. (2004). Environmental stress responses in *Lactobacillus*: a review. *Proteomics* 4 106–122. 10.1002/pmic.200300497 14730676

[B19] De Bruyne K. Camu N. De Vuyst L. Vandamme P. (2009). *Lactobacillus fabifermentans* sp. nov. and *Lactobacillus cacaonum* sp. nov., isolated from ghanaian cocoa fermentations. *Int. J. Syst. Evol. Biol.* 59 7–12. 10.1099/ijs.0.001172-0 19126714

[B20] De Taeye C. Eyamo Evina V. J. Caullet G. Niemenak N. Collin S. (2016). Fate of anthocyanins through cocoa fermentation. emergence of new polyphenolic dimers. *J. Agric. Food Chem.* 64 8876–8885. 10.1021/acs.jafc.6b03892 27934293

[B21] De Vuyst L. Leroy F. (2020). Functional role of yeasts, lactic acid bacteria, and *acetic acid bacteria* in cocoa fermentation processes. *FEMS Microbiol. Rev.* 44 432–453. 10.1093/femsre/fuaa014 32420601

[B22] De Vuyst L. Weckx S. (2016). The cocoa bean fermentation process: from ecosystem analysis to starter culture development. *J. Appl. Microbiol.* 121 5–17. 10.1111/jam.13045 26743883

[B23] Dellaglio F. Vancanneyt M. Endo A. Vandamme P. Felis G. E. Castioni A. (2006). *Lactobacillus durianis* Leisner et al. 2002 is a later heterotypic synonym of *Lactobacillus vaccinostercus* Kozaki and Okada 1983. *Int. J. Syst. Evol. Microbiol.* 56 1721–1724. 10.1099/ijs.0.64316-0 16901998

[B24] Edgar R. C. (2004). MUSCLE: multiple sequence alignment with high accuracy and high throughput. *Nucleic Acids Res.* 32 1792–1797. 10.1093/nar/gkh340 15034147 PMC390337

[B25] Emms D. M. Kelly S. (2015). OrthoFinder: solving fundamental biases in whole genome comparisons dramatically improves orthogroup inference accuracy. *Genome Biol.* 16:157. 10.1186/s13059-015-0721-2 26243257 PMC4531804

[B26] Eren A. M. Esen ÖC. Quince C. Vineis J. H. Morrison H. G. Sogin M. L. (2015). Anvi’o: an advanced analysis and visualization platform for ‘omics data. *PeerJ.* 3:e1319. 10.7717/peerj.1319 26500826 PMC4614810

[B27] Fernández Maura Y. Balzarini T. Clapé Borges P. Evrard P. De Vuyst L. Daniel H.-M. (2016). The environmental and intrinsic yeast diversity of Cuban cocoa bean heap fermentations. *Int. J. Food Microbiol.* 233 34–43. 10.1016/j.ijfoodmicro.2016.06.012 27322722

[B28] Garcia-Armisen T. Papalexandratou Z. Hendryckx H. Camu N. Vrancken G. De Vuyst L. (2010). Diversity of the total bacterial community associated with ghanaian and brazilian cocoa bean fermentation samples as revealed by a 16 S rRNA gene clone library. *Appl. Microbiol. Biotechnol.* 87 2281–2292. 10.1007/s00253-010-2698-9 20559826

[B29] Gardner J. (2016). Polysaccharide degradation systems of the saprophytic bacterium *Cellvibrio japonicus*. *World J. Microbial. Biotechnol.* 32:121. 10.1007/s11274-016-2068-6 27263016

[B30] Goffin P. Lorquet F. Kleerebezem M. Hols P. (2004). Major role of NAD-dependent lactate dehydrogenases in aerobic lactate utilization in *Lactobacillus plantarum* during early stationary phase. *J.f Bacteriol.* 186 6661–6666. 10.1128/JB.186.19.6661-6666.2004 15375150 PMC516598

[B31] Goodfellow M. Kämpfer P. Chun J. De Vos P. Rainey F. A. Whitman W. B. (2010). “Genus I. *Sphingobacterium*,” in *Bergey’s Manual of Systematic Bacteriology, Volume 4: The Bacteroidetes, Spirochaetes, Tenericutes* (*Mollicutes*), *Acidobacteria, Fibrobacteres, Fusobacteria, Dictyoglomi, Gemmatimonadetes, Lentisphaerae, Verrucomicrobia, Chlamydiae*, and *Planctomycetes*, eds Krieg N. R. Staley J. T. Brown D. R. Hedlund B. P. Paster B. J. Ward N. L. (New York: Springer-Verlag), 331–339.

[B32] Hamdouche Y. Guehi T. Durand N. Kedjebo K. B. D. Montet D. Meile J. C. (2015). Dynamics of microbial ecology during cocoa fermentation and drying: towards identification of molecular markers. *Food Control* 48 117–122. 10.1016/j.foodcont.2014.05.031

[B33] Ho V. T. T. Fleet G. H. Zhao J. (2018). Unravelling the contribution of lactic acid bacteria and *acetic acid bacteria* to cocoa fermentation using inoculated organisms. *Int. J. Food Microbiol.* 279 43–56. 10.1016/j.ijfoodmicro.2018.04.040 29727857

[B34] Ho V. T. T. Zhao J. Fleet G. (2014). Yeasts are essential for cocoa bean fermentation. *Int. J. Food Microbiol.* 174 72–87. 10.1016/j.ijfoodmicro.2013.12.014 24462702

[B35] Ho V. T. T. Zhao J. Fleet G. (2015). The effect of lactic acid bacteria on cocoa bean fermentation. *Int. J. Food Microbiol.* 205 54–67. 10.1016/j.ijfoodmicro.2015.03.031 25889523

[B36] Holm C. S. Aston J. W. Douglas K. (1993). The effects of the organic acids in cocoa on the flavor of chocolate. *J. Sci. Food Agric.* 61 65–71. 10.1002/jsfa.2740610111

[B37] Holt C. Yandell M. (2011). MAKER2: an annotation pipeline and genome-database management tool for second-generation genome projects. *BMC Bioinfor.* 12:491. 10.1186/1471-2105-12-491 22192575 PMC3280279

[B38] Hugouvleux-Cotte-Pattat N. Condemine G. Shevchik V. (2014). Bacterial pectate lyases, structural and functional diversity. *Environ. Microbiol. Rep.* 6 427–440. 10.1111/1758-2229.12166 25646533

[B39] Huson D. H. Beier S. Flade I. Górska A. El-Hadidi M. Mitra S. (2016). MEGAN Community Edition – interactive exploration and analysis of large-scale microbiome sequencing data. *PLoS Comp. Biol.* 12:e1004957. 10.1371/journal.pcbi.1004957 27327495 PMC4915700

[B40] Illeghems K. De Vuyst L. Papalexandratou Z. Weckx S. (2012). Phylogenetic analysis of a spontaneous cocoa bean fermentation metagenome reveals new insights into its bacterial and fungal community diversity. *PLoS One* 7:e34080. 10.1371/journal.pone.0038040 22666442 PMC3362557

[B41] Illeghems K. Weckx S. De Vuyst L. (2015). Applying meta-pathway analyses through metagenomics to identify the functional properties of the major bacterial communities of a single spontaneous cocoa bean fermentation process sample. *Food Microbiol.* 50 54–63. 10.1016/j.fm.2015.03.005 25998815

[B42] Jamili J. Yanti N. A. Susilowati P. E. (2016). Diversity and the role of yeast in spontaneous cocoa bean fermentation from southeast sulawesi, indonesia. *Biodiversitas* 17 90–95. 10.13057/biodiv/d170113

[B43] Jespersen L. Nielsen D. S. Hønholt S. Jakobsen M. (2005). Occurrence and diversity of yeasts involved in fermentation of west african cocoa beans. *FEMS Yeast Res.* 5 441–453. 10.1016/j.femsyr.2004.11.002 15691749

[B44] Kämpfer P. (2012). “Genus I. *Streptomyces*,” in *Bergey’s Manual of Systematic Bacteriology, Volume 5: The Actinobacteria*, eds Whitman W. Goodfellow M. Kämpfer P. Busse H.-J. Trujillo M. Ludwig W. (New York: Springer-Verlag), 1455–1767.

[B45] Koné M. K. Guéhi S. T. Durand N. Ban-Koffi L. Berthiot L. Fontana Tachon A. (2016). Contribution of predominant yeasts to the occurrence of aroma compounds during cocoa bean fermentation. *Food Res. Int.* 89 910–917. 10.1016/j.foodres.2016.04.010

[B46] Kopylova E. Noé L. Touzet H. (2012). SortMeRNA: fast and accurate filtering of ribosomal RNAs in metatranscriptomic data. *Bioinformatics* 28 3211–3217. 10.1093/bioinformatics/bts611 23071270

[B47] Kube M. Siewert C. Migdoll A. M. Duduk B. Holz S. Rabus R. (2014). Analysis of the complete genomes of *Acholeplasma brassicae*, *A. palmae* and *A. laidlawii* and their comparison to the obligate parasites from ‘*Candidatus* Phytoplasma’. *J. Mol. Microbiol. Biotechnol.* 24 19–36. 10.1159/000354322 24158107

[B48] Langmead B. Salzberg S. (2012). Fast gapped-read alignment with Bowtie 2. *Nat. Methods* 9 357–359. 10.1038/nmeth.1923 22388286 PMC3322381

[B49] Lawrence N. L. Wilson D. C. Pederson C. S. (1959). The growth of yeasts in grape juice stored at low temperatures. II. The types of yeasts and their growth in pure culture. *Appl. Microbiol.* 7 7–11. 10.1128/aem.7.1.7-11.1959 13617938 PMC1057453

[B50] Lefeber T. Gobert W. Vrancken G. Camu N. De Vuyst L. (2011). Dynamics and species diversity of communities of lactic acid bacteria and *acetic acid bacteria* during spontaneous cocoa bean fermentation in vessels. *Food Microbiol.* 28 457–464. 10.1016/j.fm.2010.10.010 21356451

[B51] Lefeber T. Janssens M. Camu N. De Vuyst L. (2010). Kinetic analysis of strains of lactic acid bacteria and *acetic acid bacteria* in cocoa pulp simulation media toward development of a starter culture for cocoa bean fermentation. *Appl. Environ. Microbiol.* 76 7708–7716. 10.1128/AEM.01206-10 20889778 PMC2988588

[B52] Lefeber T. Papalexandratou Z. Gobert W. Camu N. De Vuyst L. (2012). On-farm implementation of a starter culture for improved cocoa bean fermentation and its influence on the flavour of chocolates produced thereof. *Food Microbiol.* 30 379–392. 10.1016/j.fm.2011.12.021 22365351

[B53] Li D. H. Liu C. M. Luo R. B. Sadakane K. Lam T. W. (2015). MEGAHIT: an ultra-fast single-node solution for large and complex metagenomics assembly via succinct de Bruijn graph. *Bioinformatics* 31 1674–1676. 10.1093/bioinformatics/btv033 25609793

[B54] Li H. (2018). Minimap2: pairwise alignment for nucleotide sequences. *Bioinformatics* 34 3094–3100. 10.1093/bioinformatics/bty191 29750242 PMC6137996

[B55] Lima C. O. Vaz A. B. M. De Castro G. M. Lobo F. Solar R. Rodrigues C. (2020). Integrating microbial metagenomics and physicochemical parameters and a new perspective on starter culture for fine cocoa fermentation. *Food Microbiol*. 93:103608. 10.1016/j.fm.2020.103608 32912581

[B56] Magalhães da Veiga Moreira I. da Cruz Pedroso Miguel M. G. Lacerda Ramos C. Ferreira Duarte W. Efraim P. Freitas Schwan R. (2016). Influence of cocoa hybrids on volatile compounds of fermented beans, microbial diversity during fermentation and sensory characteristics and acceptance of chocolates. *J. Food Qual.* 39 839–849. 10.1111/jfq.12238

[B57] Marchler-Bauer A. Bo Y. Han L. He J. Lanczycki C. J. Lu S. (2017). CDD/SPARCLE: functional classification of proteins via subfamily domain architectures. *Nucleic Acids Res.* 45 D200–D203. 10.1093/nar/gkw1129 27899674 PMC5210587

[B58] Mbenoun M. Wingfield M. J. Begoude Boyogueno A. D. Nsouga Amougou F. Petchayo Tigang S. Ten Hoopen G. M. (2016). Diversity and pathogenicity of the *Ceratocystidaceae* associated with cacao agroforests in cameroon. *Plant Pathol.* 65 64–78. 10.1111/ppa.12400

[B59] Meersman E. Steensels J. Paulus T. Struyf N. Saels V. Mathawan M. (2015). Breeding strategy to generate robust yeast starter cultures for cocoa pulp fermentations. *Appl. Environ. Microbiol.* 81 6166–6176. 10.1128/AEM.00133-15 26150457 PMC4542255

[B60] Meersman E. Struyf N. Kyomugasho C. Jamsazzadeh Kermani Z. Santiago J. S. Baert E. (2017). Characterization and degradation of pectic polysaccharides in cocoa pulp. *J. Agric. Food Chem.* 65 9726–9734. 10.1021/acs.jafc.7b03854 29032689

[B61] Mejía L. C. Rojas E. I. Maynard Z. Van Bael S. Arnold A. E. Hebbar P. (2008). Endophytic fungi as biocontrol agents of *Theobroma cacao* pathogens. *Biol. Control* 46 4–14. 10.1016/j.biocontrol.2008.01.012

[B62] Menzel P. Ng K. L. Krogh A. (2016). Fast and sensitive taxonomic classification for metagenomics with Kaiju. *Nat.Commun.* 7:11257. 10.1038/ncomms11257 27071849 PMC4833860

[B63] Mitchell P. L. (2004). Heteroptera as vectors of plant pathogens. *Neotropical. Entomol.* 33 519–545. 10.1590/S1519-566X2004000500001

[B64] Moens F. Lefeber T. De Vuyst L. (2014). Oxidation of metabolites highlights the microbial interactions and role of *Acetobacter pasteurianus* during cocoa bean fermentation. *Appl. Environ. Microbiol.* 80 1848–1857. 10.1128/AEM.03344-13 24413595 PMC3957632

[B65] Muñoz M. S. Cortina J. R. Vaillant F. E. Parra S. E. (2020). An overview of the physical and biochemical transformation of cocoa seeds to beans and to chocolate: flavor formation. *Crit. Rev. Food Sci. Nutrition* 60 1593–1613. 10.1080/10408398.2019.1581726 30896305

[B66] Murphy M. G. O’Connor L. Walsh D. Condon S. (1985). Oxygen dependent lactate utilization by *Lactobacillus plantarum*. *Arch. Microbiol.* 141 75–79. 10.1007/BF00446743 3994484

[B67] NCBI Resource Coordinators. (2016). Database resources of the national center for biotechnology information. *Nucleic Acids Res.* 44 D7–D19. 10.1093/nar/gkv1290 26615191 PMC4702911

[B68] Nielsen D. S. Teniola O. D. Ban-Koffi L. Owusu M. Andersson T. S. Holzapfel W. H. (2007). The microbiology of Ghanaian cocoa fermentations analysed using culture-dependent and culture-independent methods. *Int. J. Food Microbiol.* 114 168–186. 10.1016/j.ijfoodmicro.2006.09.010 17161485

[B69] Oksanen J. Blanchet F. G. Friendly M. Kindt R. Legendre P. McGlinn D. (2019). *Vegan: community ecology package. R package version 2.5-6.* Available Online at: https://CRAN.R-project.org/package=vegan (accessed November 3, 2020).

[B70] O’Leary N. A. Wright M. W. Brister J. R. Ciufo S. Haddad D. McVeigh R. (2016). Reference sequence (RefSeq) database at NCBI: current status, taxonomic expansion, and functional annotation. *Nucleic Acids Res.* 44 D733–D745. 10.1093/nar/gkv1189 26553804 PMC4702849

[B71] Ouattara H. D. Ouattara H. G. Droux M. Reverchon S. Nasser W. Niamke S. L. (2017). Lactic acid bacteria involved in cocoa beans fermentation from ivory coast: species diversity and citrate lyase production. *Int. J. Food Microbiol.* 256 11–19. 10.1016/j.ijfoodmicro.2017.05.008 28578265

[B72] Ouattara H. G. Elias R. J. Dudley E. (2020). Microbial synergy between *Pichia kudriavzevii* YS201 and *Bacillus subtilis* BS38 improves pulp degradation and aroma production in cocoa pulp simulation medium. *Heliyon* 6:e03269. 10.1016/j.heliyon.2020.e03269 31993527 PMC6971349

[B73] Ouattara H. G. Koffi B. L. Karou G. T. Sangaré A. Niamke S. L. Diopoh J. K. (2008). Implication of *Bacillus* sp. in the production of pectinolytic enzymes during cocoa fermentation. *World J. Microbial. Biotechnol.* 24 1753–1760. 10.1007/s11274-008-9683-9

[B74] Papadimitriou K. Alegría A. Bron P. A. de Angelis M. Gobbetti M. Kleerebezem M. (2016). Stress physiology of lactic acid bacteria. *Microbiol. Mol. Biol.Rev.* 80 837–890. 10.1128/MMBR.00076-15 27466284 PMC4981675

[B75] Papalexandratou Z. Camu N. Falony G. De Vuyst L. (2011a). Comparison of the bacterial species diversity of spontaneous cocoa bean fermentations carried out at selected farms in Ivory Coast and Brazil. *Food Microbiol.* 28 964–973. 10.1016/j.fm.2011.01.010 21569940

[B76] Papalexandratou Z. Falony G. Romanens E. Jimenez J. C. Amores F. Daniel H.-M. (2011b). Species diversity, community dynamics, and metabolite kinetics of the microbiota associated with traditional Ecuadorian spontaneous cocoa bean fermentations. *Appl. Environ. Microbiol.* 77 7698–7714. 10.1128/AEM.05523-11 21926224 PMC3209185

[B77] Papalexandratou Z. Vrancken G. De Bruyne K. Vandamme P. De Vuyst L. (2011c). Spontaneous organic cocoa bean box fermentations in brazil are characterized by a restricted species diversity of lactic acid bacteria and *acetic acid bacteria*. *Food Microbiol.* 28 1326–1338. 10.1016/j.fm.2011.06.003 21839382

[B78] Papalexandratou Z. De Vuyst L. (2011). Assessment of the yeast species composition of cocoa bean fermentations in different cocoa-producing regions using denaturing gradient gel electrophoresis. *FEMS Yeast Res.* 11 564–574. 10.1111/j.1567-1364.2011.00747.x 22093683

[B79] Papalexandratou Z. Kaasik K. Villagra Kaufmann L. Skorstengaard A. Bouillon G. Leth Espensen J. (2019). Linking cocoa varietals and microbial diversity of Nicaraguan fine cocoa bean fermentations and their impact on final cocoa quality appreciation. *Int. J. Food Microbiol.* 304 106–118. 10.1016/j.ijfoodmicro.2019.05.012 31176963

[B80] Papalexandratou Z. Lefeber T. Bahrim B. Lee O. S. Daniel H.-M. De Vuyst L. (2013). *Hanseniaspora opuntiae, Saccharomyces cerevisiae*, *Lactobacillus fermentum*, and *Acetobacter pasteurianus* predominate during well-performed malaysian cocoa bean box fermentations, underlining the importance of these microbial species for a successful cocoa bean fermentation process. *Food Microbiol.* 35 73–85. 10.1016/j.fm.2013.02.015 23664257

[B81] Parks D. H. Imelfort M. Skennerton C. T. Hugenholtz P. Tyson G. W. (2015). CheckM: assessing the quality of microbial genomes recovered from isolates, single cells, and metagenomes. *Genome Res.* 25 1043–1055. 10.1101/gr.186072.114 25977477 PMC4484387

[B82] Petersen T. N. Brunak S. von Heijne G. Nielsen H. (2011). SignalP 4.0: discriminating signal peptides from transmembrane regions. *Nat. Methods* 8 785–786. 10.1038/nmeth.1701 21959131

[B83] Rademacher C. Masepohl B. (2012). Copper-responsive gene regulation in bacteria. *Microbiology* 158 2451–2464. 10.1099/mic.0.058487-0 22918892

[B84] Samagaci L. Ouattara H. Niamké S. Lemaire M. (2016). *Pichia kudriavzevii* and *Candida nitrativorans* are the most well-adapted and relevant yeast species fermenting cocoa in agneby-tiassa, a local Ivorian cocoa producing region. *Food Res. Int.* 89 773–780. 10.1016/j.foodres.2016.10.007 28460978

[B85] Sanchez Y. Lindquist S. L. (1990). HSP104 required for induced thermotolerance. *Science* 248 1112–1115. 10.1126/science.2188365 2188365

[B86] Schmieder R. Edwards R. (2011). Quality control and preprocessing of metagenomic datasets. *Bioinformatics* 27 863–864. 10.1093/bioinformatics/btr026 21278185 PMC3051327

[B87] Schwan R. F. Cooper R. M. Wheals A. E. (1997). Endopolygalacturonase secretion by *Kluyveromyces marxianus* and other cocoa pulp-degrading yeasts. *Enzyme Microbial. Technol.* 21 234–244. 10.1016/S0141-0229(96)00261-X

[B88] Schwan R. F. Wheals A. E. (2004). The microbiology of cocoa fermentation and its role in chocolate quality. *Crit. Rev. Food Sci. Nutrition* 44 205–221. 10.1080/10408690490464104 15462126

[B89] Sedewitz B. Schleifer K. H. Götz F. (1984). Physiological role of pyruvate oxidase in the aerobic metabolism of *Lactobacillus plantarum*. *J. Bacteriol.* 160 462–465. 10.1128/jb.160.1.462-465.1984 6480562 PMC214746

[B90] Seemann T. (2014). Prokka: rapid prokaryotic genome annotation. *Bioinformatics* 30 2068–2069. 10.1093/bioinformatics/btu153 24642063

[B91] Serra J. L. Moura F. G. de Melo Pereira G. V. Soccol C. R. Rogez H. Darnet S. (2019). Determination of the microbial community in amazonian cocoa bean fermentation by Illumina-based metagenomic sequencing. *LWT – Food Sci. Technol.* 106 229–239. 10.1016/j.lwt.2019.02.038

[B92] Shah N. Nute M. G. Warnow T. Pop M. (2019). Misunderstood parameter of NCBI BLAST impacts the correctness of bioinformatics workflows. *Bioinformatics* 35 1613–1614. 10.1093/bioinformatics/bty833 30247621

[B93] The UniProt Consortium. (2019). UniProt: a worldwide hub of protein knowledge. *Nucleic Acids Res.* 47 D506–D515. 10.1093/nar/gky1049 30395287 PMC6323992

[B94] Truong D. T. Franzosa E. A. Tickle T. L. Scholz M. Weingart G. Pasolli E. (2015). MetaPhlAn2 for enhanced metagenomic taxonomic profiling. *Nat. Methods* 12 902–903. 10.1038/nmeth.3589 26418763

[B95] van den Brink J. de Vries R. P. (2011). Fungal enzyme sets for plant polysaccharide degradation. *Appl. Microbiol. Biotechnol.* 91 1477–1492. 10.1007/s00253-011-3473-2 21785931 PMC3160556

[B96] Vaughan-Martini A. Martini A. (2011). “*Saccharomyces* Meyen ex Reess (1870),” in *The Yeasts*, Fifth Edn, eds Kurtzman C. P. Fell J. W. Boekhout T. (Netherland: Elsevier), 733–746. 10.1016/b978-0-444-52149-1.00061-6

[B97] Verce M. De Vuyst L. Weckx S. (2019). Shotgun metagenomics of a water kefir fermentation ecosystem reveals a novel *Oenococcus* species. *Front. Microbiol.* 10:479. 10.3389/fmicb.2019.00479 30918501 PMC6424877

[B98] Vermote L. Verce M. De Vuyst L. Weckx S. (2018). Amplicon and shotgun metagenomic sequencing indicates that microbial ecosystems present in cheese brines reflect environmental inoculation during the cheese production process. *Int. Dairy J.* 87 44–53. 10.1016/j.idairyj.2018.07.010

[B99] Visintin S. Alessandria V. Valente A. Dolci P. Cocolin L. (2016). Molecular identification and physiological characterization of yeasts, lactic acid bacteria and *acetic acid bacteria* isolated from heap and box cocoa bean fermentations in west africa. *Int. J. Food Microbiol.* 216 69–78. 10.1016/j.ijfoodmicro.2015.09.004 26425801

[B100] Voigt J. Heinrichs H. Voigt G. Biehl B. (1994). Cocoa-specific aroma precursors are generated by proteolytic digestion of vicilin-like globulin of cocoa seeds. *Food Chem.* 50 177–184. 10.1016/0308-8146(94)90117-1

[B101] Wollgast J. Anklam E. (2000). Review of polyphenols in *Theobroma cacao*: changes in composition during the manufacture of chocolate and methodology for identification and quantification. *Food Res. Int.* 33 423–447. 10.1016/S0963-9969(00)00068-5

[B102] Wood D. E. Lu J. Langmead B. (2019). Improved metagenomic analysis with Kraken 2. *Genome Biol.* 20:257. 10.1186/s13059-019-1891-0 31779668 PMC6883579

[B103] Yamada Y. Yukphan P. Vu H. T. L. Muramatsu Y. Ochaikul D. Tanasupawat S. (2012). Description of *Komagataeibacter* gen. nov., with proposals of new combinations (*Acetobacteraceae*). *J. General Appl. Microbiol.* 58 397–404. 10.2323/jgam.58.397 23149685

[B104] Zaunmüller T. Eichert M. Richter H. Unden G. (2006). Variations in the energy metabolism of biotechnologically relevant heterofermentative lactic acid bacteria during growth on sugars and organic acids. *Appl. Microbiol. Biotechnol.* 72 421–429. 10.1007/s00253-006-0514-3 16826375

[B105] Zhang H. Yohe T. Huang L. Entwistle S. Wu P. Yang Z. (2018). dbCAN2: a meta server for automated carbohydrate-active enzyme annotation. *Nucleic Acids Res.* 46 W95–W101. 10.1093/nar/gky418 29771380 PMC6031026

[B106] Zhang S. J. De Bruyn F. Pothakos V. Torres J. Falconi C. Moccand C. (2019). Following coffee production from cherries to cup: microbiological and metabolomic analysis of wet processing of *Coffea arabica*. *Appl. Environ. Microbiol.* 85 e2318–e2365. 10.1128/AEM.02635-18 30709820 PMC6414394

[B107] Zheng J. Wittouck S. Salvetti E. Franz C. M. A. P. Harris H. M. B. Mattarelli P. (2020). A taxonomic note on the genus *Lactobacillus*: description of 23 novel genera, emended description of the genus *Lactobacillus* Beijerinck 1901, and union of *Lactobacillaceae* and *Leuconostocaceae*. *Int. J. Syst. Evol. Bacteriol.* 70 2782–2858. 10.1099/ijsem.0.004107 32293557

