# Supplementary material for: A Combined Metagenomics and Metatranscriptomics Approach to Unravel Costa Rican Cocoa Box Fermentation Processes Reveals Yet Unreported Microbial Species and Functionalities
Source: Front Microbiol. 2021 Feb 16;12:641185. doi: 10.3389/fmicb.2021.641185 (PMC7920976; doi:10.3389/fmicb.2021.641185)

Supplementary Figure 1. Temporal dynamics of **(A)** the major microbial genera and **(B)** the minor microbial genera, based on an overall taxonomic analysis of metagenomic data sets of the Costa Rican cocoa box fermentation processes (F1, F2 and F3). The genera were categorised into groups indicated by different colours, based on the phase of the box fermentation process wherein they were assigned the most reads: B – beginning of the box fermentation process, BM – beginning and middle of the box fermentation process, M – middle of the box fermentation process, E – end of the box fermentation process, BE – beginning and end of the box fermentation process. Full lines – box fermentation process F2, dotted lines – box fermentation process F1, dashed lines – box fermentation process F3.

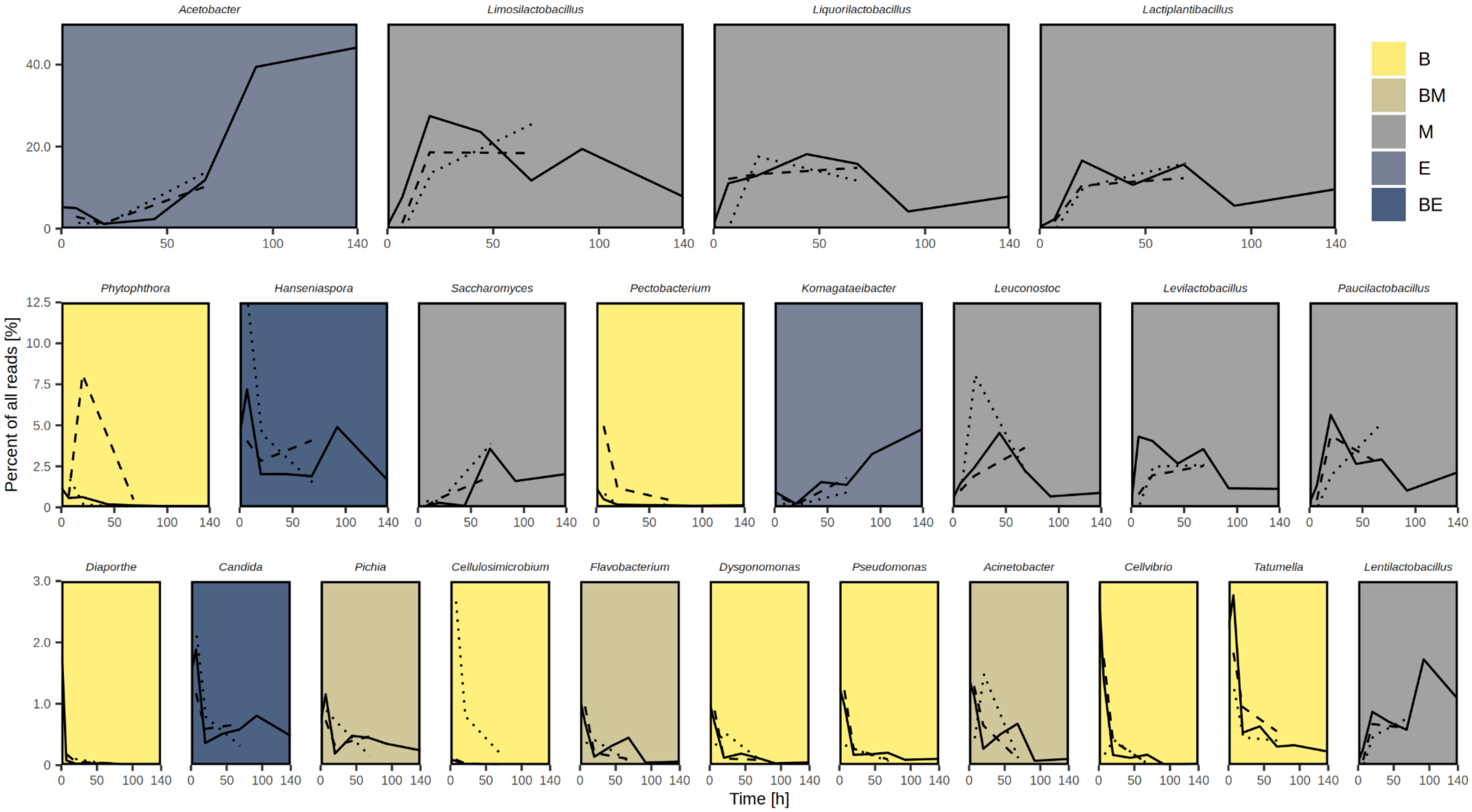

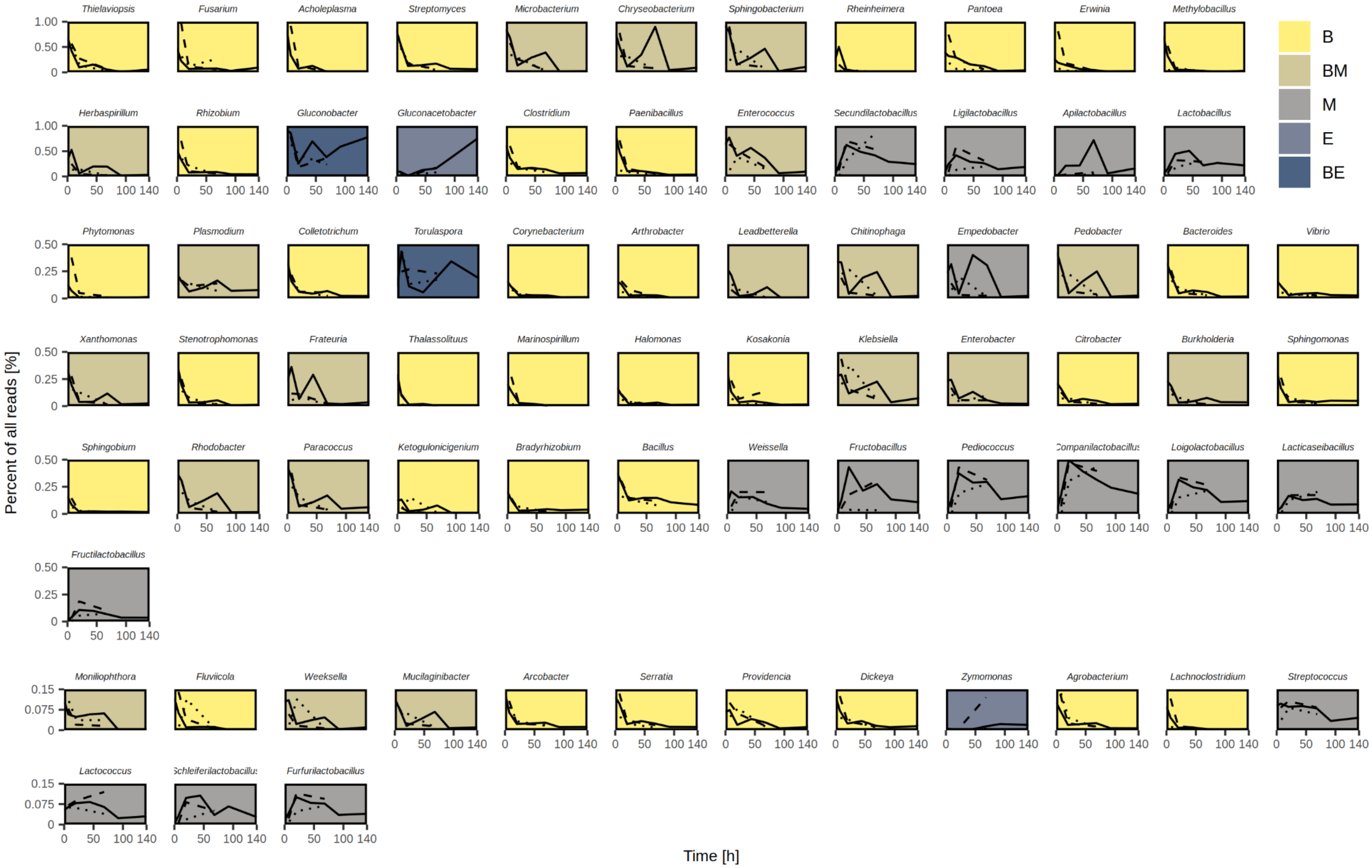

Thielaviopsis
Fusarium
Acholeplasma
Streptomyces
Microbacterium
Chryseobacterium
Sphingobacterium
Rheinheimera
Pantoea
Erwinia
Methylobacillus
Herbaspirillum
Rhizobium
Gluconobacter
Gluconacetobacter
Clostridium
Paenibacillus
Enterococcus
Secundilactobacillus
Ligilactobacillus
Apilactobacillus
Lactobacillus
Phytomonas
Plasmodium
Colletotrichum
Torulaspora
Corynebacterium
Arthrobacter
Leadbetterella
Chitinophaga
Empedobacter
Pedobacter
Bacteroides
Vibrio
Xanthomonas
Stenotrophomonas
Frateuria
Thalassolituus
Marinospirillum
Halomonas
Kosakonia
Klebsiella
Enterobacter
Citrobacter
Burkholderia
Sphingomonas
Sphingobium
Rhodobacter
Paracoccus
Ketogulonicigenium
Bradyrhizobium
Bacillus
Weissella
Fructobacillus
Pediococcus
Companilactobacillus
Loigolactobacillus
Lacticaseibacillus
Fructilactobacillus
Moniliophthora
Fluviicola
Weeksella
Mucilaginibacter
Arcobacter
Serratia
Providencia
Dickeya
Zymomonas
Agrobacterium
Lachnoclostridium
Streptococcus
Lactococcus
Schleiferilactobacillus
Furfurilactobacillus
B
BM
M
E
BE
Percent of all reads [%]
Time [h]
0
50
100
140
0.50
0.25
1.00
0.15
0.075

Supplementary Figure 2. Metagenomic recruitment plots of species identified as present or species whose close relatives without available genome sequences were present in the Costa Rican cocoa box fermentation processes F1, F2, or F3. Recruitment plots were made for each species based on the data sets with the highest number of reads recruited where presence could be concluded.

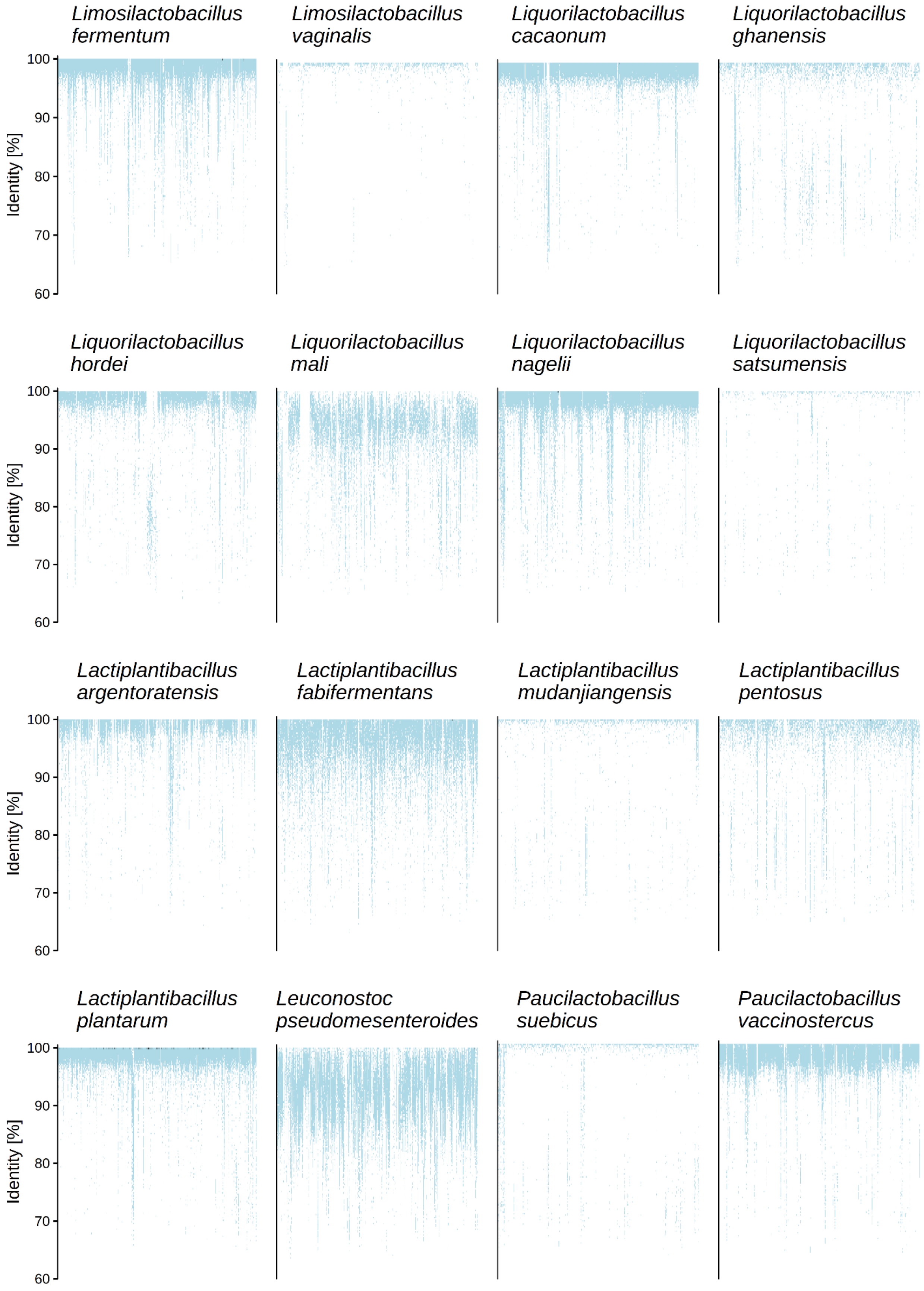

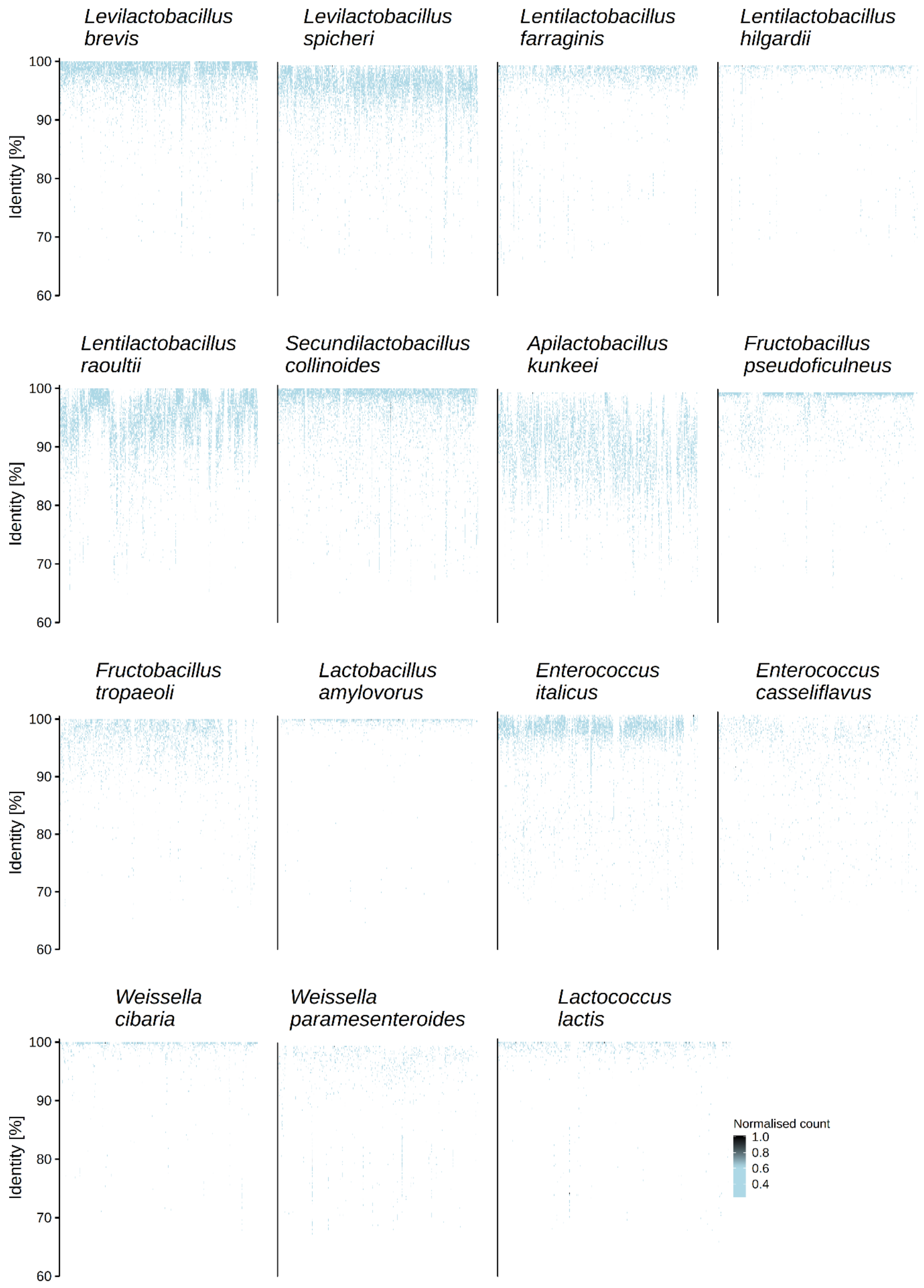
Levilactobacillus brevis
Levilactobacillus spicheri
Lentilactobacillus farraginis
Lentilactobacillus hilgardii
Lentilactobacillus raoultii
Secundilactobacillus collinoides
Apilactobacillus kunkeei
Fructobacillus pseudoficulneus
Fructobacillus tropaeoli
Lactobacillus amylovorus
Enterococcus italicus
Enterococcus casseliflavus
Weissella cibaria
Weissella paramesenteroides
Lactococcus lactis
Identity [%]
100
90
80
70
60
Normalised count
1.0
0.8
0.6
0.4

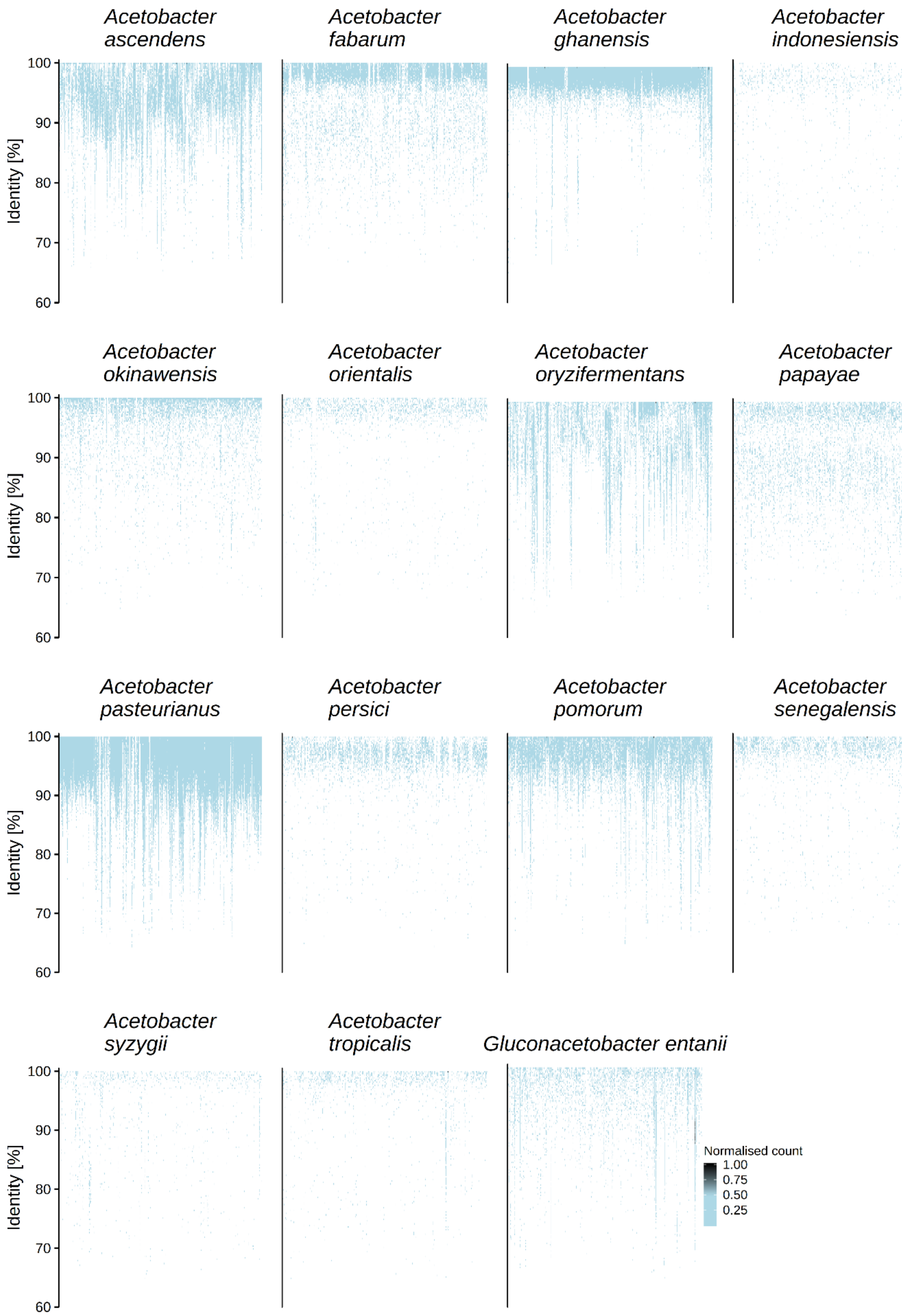
Acetobacter ascendens
Acetobacter fabarum
Acetobacter ghanensis
Acetobacter indonesiensis
Acetobacter okinawensis
Acetobacter orientalis
Acetobacter oryzifermentans
Acetobacter papayae
Acetobacter pasteurianus
Acetobacter persici
Acetobacter pomorum
Acetobacter senegalensis
Acetobacter syzygii
Acetobacter tropicalis
Gluconacetobacter entanii
Identity [%]
100
90
80
70
60
Normalised count
1.00
0.75
0.50
0.25

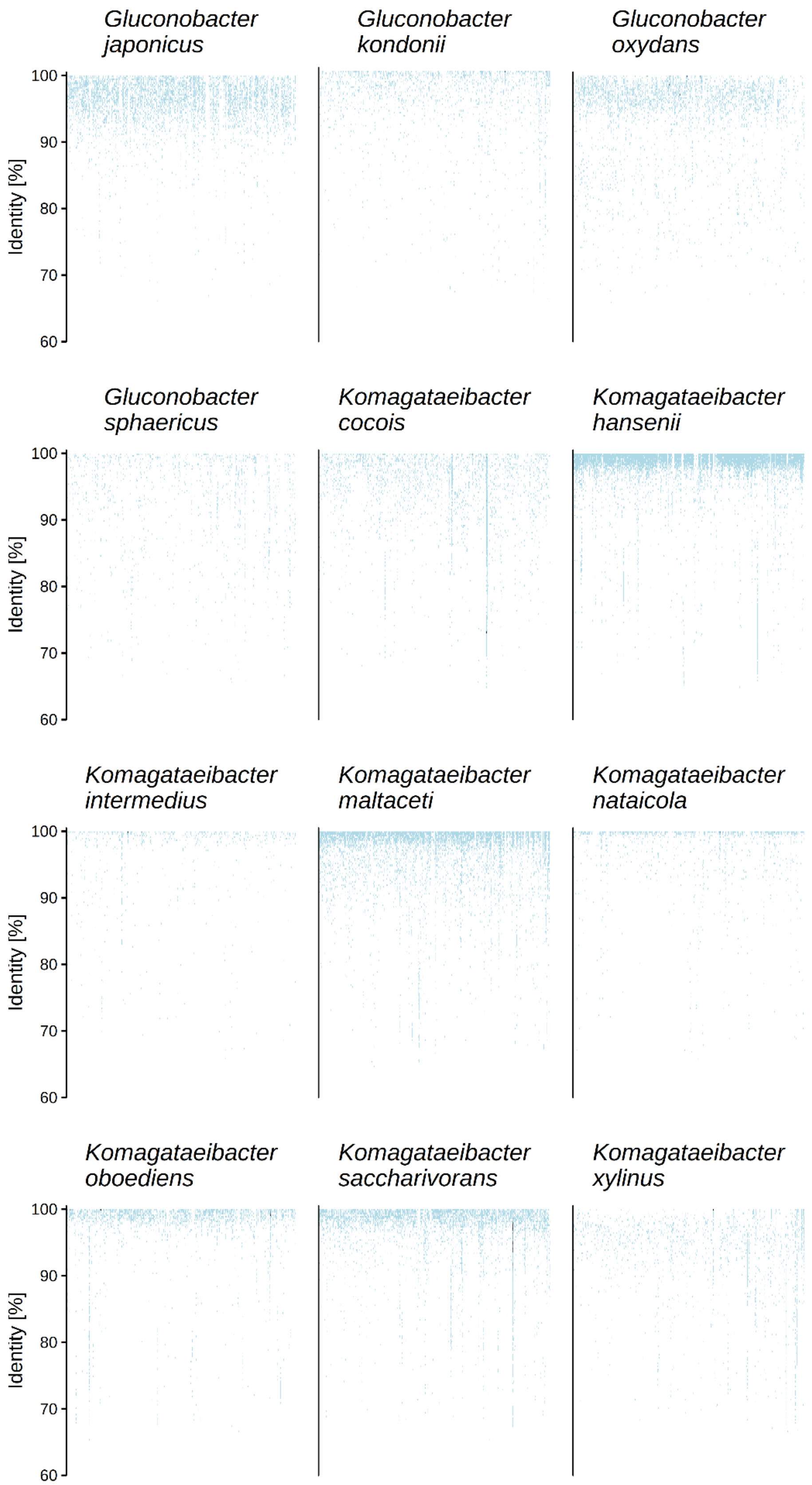
Gluconobacter japonicus
Gluconobacter kondonii
Gluconobacter oxydans
Gluconobacter sphaericus
Komagataeibacter cocois
Komagataeibacter hansenii
Komagataeibacter intermedius
Komagataeibacter maltaceti
Komagataeibacter nataicola
Komagataeibacter oboediens
Komagataeibacter saccharivorans
Komagataeibacter xylinus
Identity [%]
100
90
80
70
60

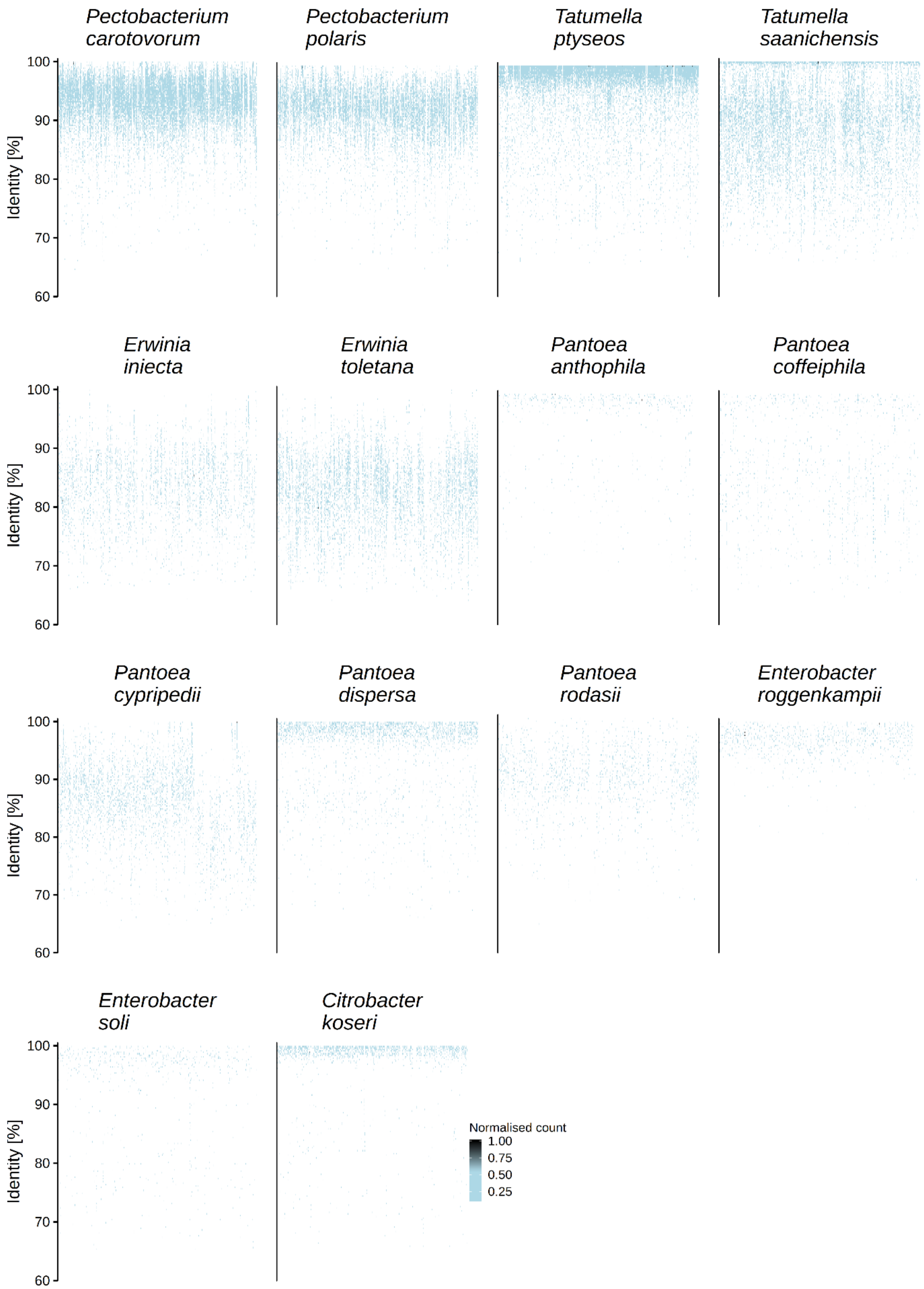

Pectobacterium carotovorum
Pectobacterium polaris
Tatumella ptyseos
Tatumella saanichensis
Erwinia iniecta
Erwinia toletana
Pantoea anthophila
Pantoea coffeiphila
Pantoea cypripedii
Pantoea dispersa
Pantoea rodasii
Enterobacter roggenkampii
Enterobacter soli
Citrobacter koseri
Identity [%]
100
90
80
70
60
Normalised count
1.00
0.75
0.50
0.25

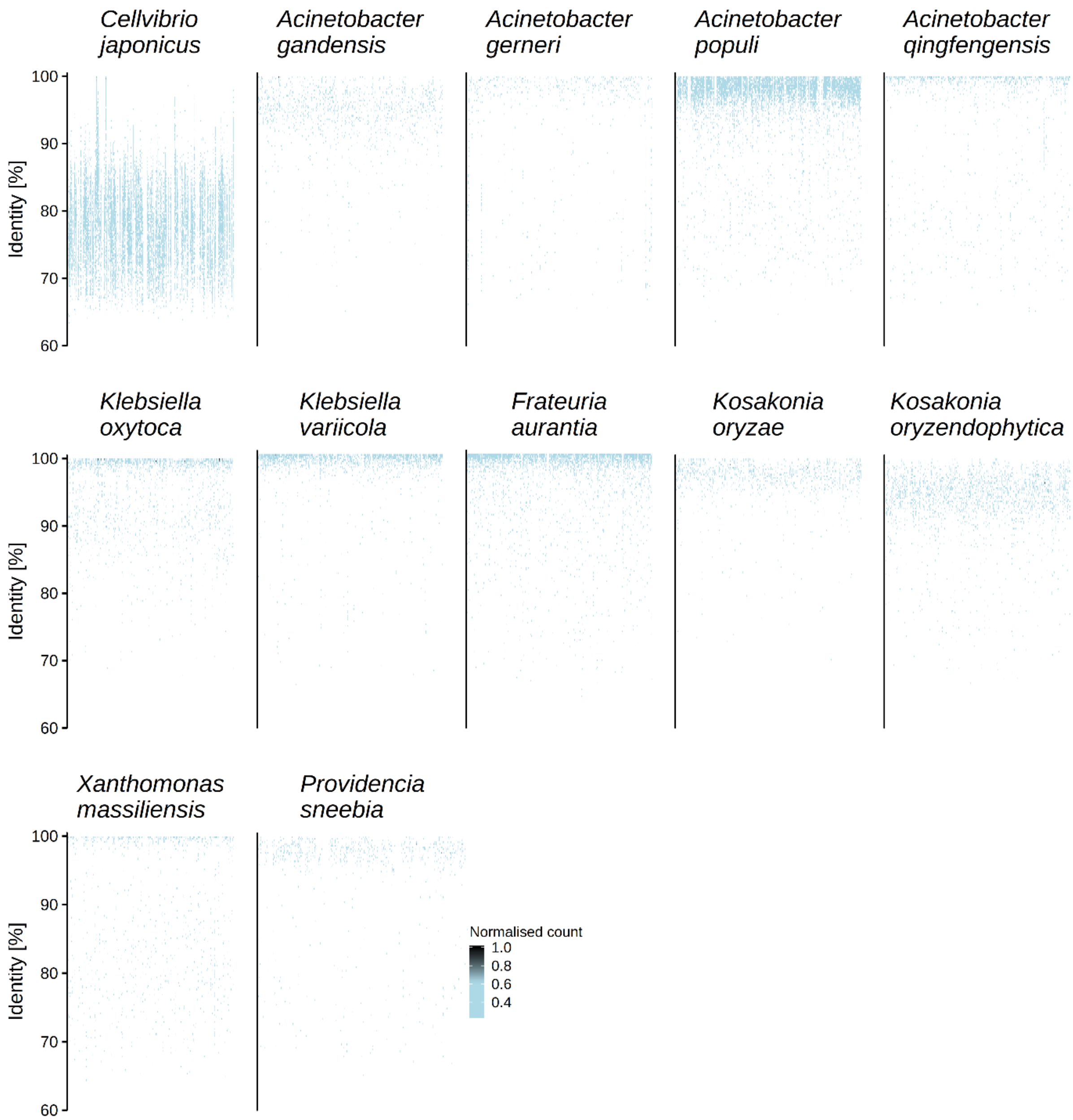

Cellvibrio japonicus
Acinetobacter gandensis
Acinetobacter gerneri
Acinetobacter populi
Acinetobacter qingfengensis
Klebsiella oxytoca
Klebsiella variicola
Frateuria aurantia
Kosakonia oryzae
Kosakonia oryzendophytica
Xanthomonas massiliensis
Providencia sneebia
Identity [%]
100
90
80
70
60
Normalised count
1.0
0.8
0.6
0.4

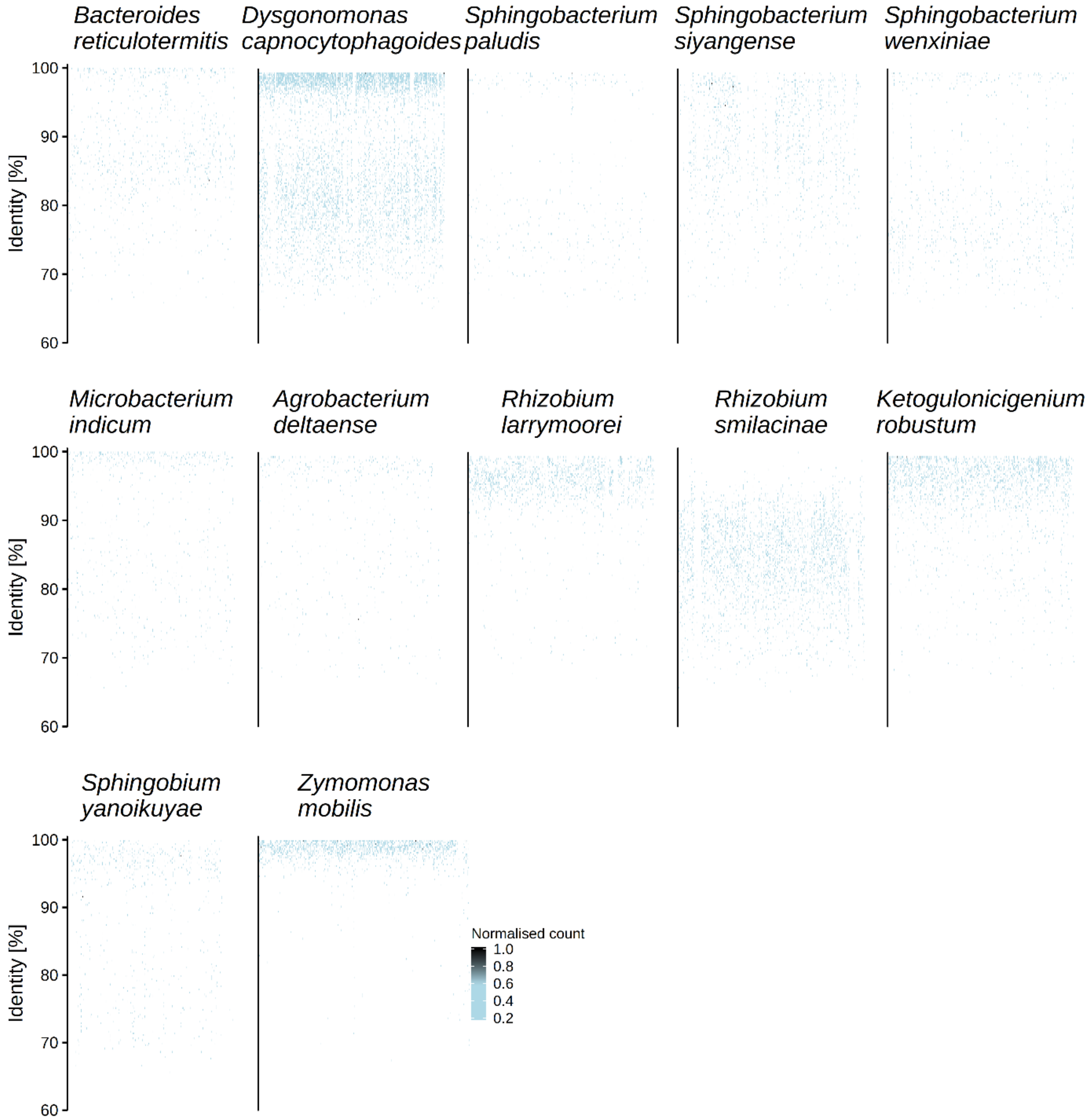

Bacteroides reticulotermitis
Dysgonomonas capnocytophagoides
Sphingobacterium paludis
Sphingobacterium siyangense
Sphingobacterium wenxiniae
Microbacterium indicum
Agrobacterium deltaense
Rhizobium larrymoorei
Rhizobium smilacinae
Ketogulonicigenium robustum
Sphingobium yanoikuyae
Zymomonas mobilis
Identity [%]
100
90
80
70
60
Normalised count
1.0
0.8
0.6
0.4
0.2

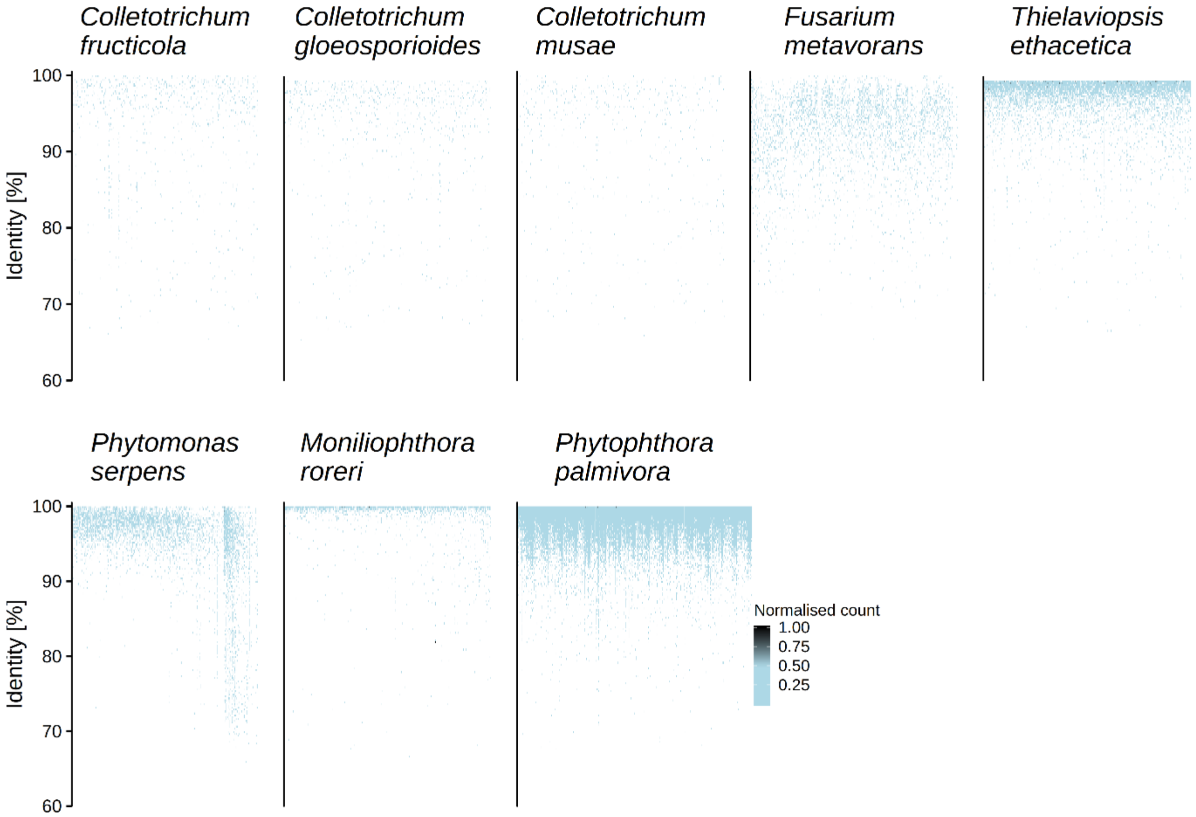
Colletotrichum fructicola
Colletotrichum gloeosporioides
Colletotrichum musae
Fusarium metavorans
Thielaviopsis ethacetica
Phytomonas serpens
Moniliophthora roreri
Phytophthora palmivora
Identity [%]
100
90
80
70
60
Normalised count
1.00
0.75
0.50
0.25

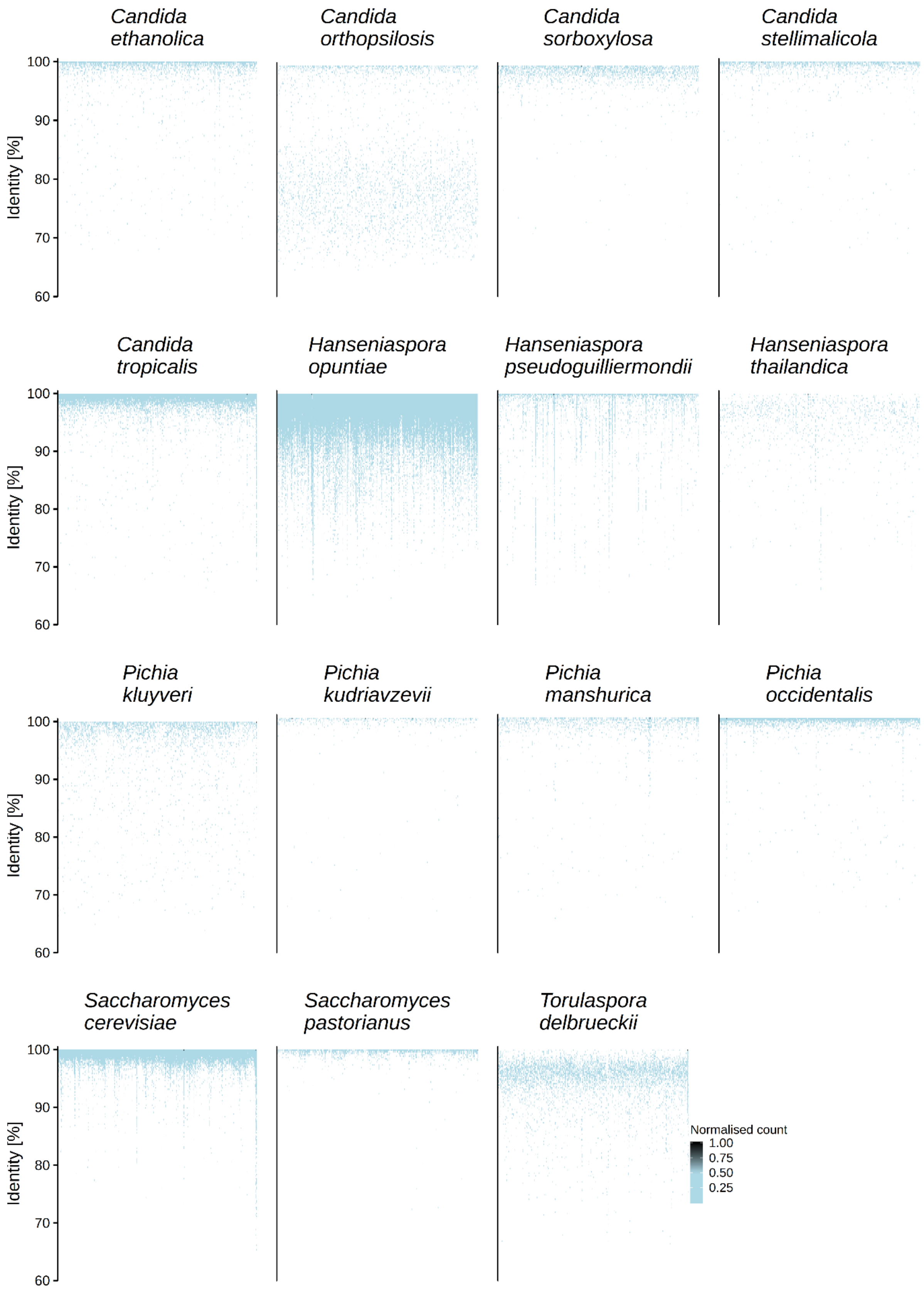

Candida ethanolica
Candida orthopsilosis
Candida sorboxylosa
Candida stellimalicola
Candida tropicalis
Hanseniaspora opuntiae
Hanseniaspora pseudoguilliermondii
Hanseniaspora thailandica
Pichia kluyveri
Pichia kudriavzevii
Pichia manshurica
Pichia occidentalis
Saccharomyces cerevisiae
Saccharomyces pastorianus
Torulaspora delbrueckii
Identity [%]
100
90
80
70
60
Normalised count
1.00
0.75
0.50
0.25

Supplementary Figure 3. Concentrations of organic acids, short-chain fatty acids, and volatile organic compounds measured in the cocoa pulp (P) and cocoa beans (B) during the Costa Rican cocoa box fermentation processes F1, F2, and F3 that were found in low concentrations with stable trends. The concentrations were determined in triplicate. The error bars represent standard deviations.

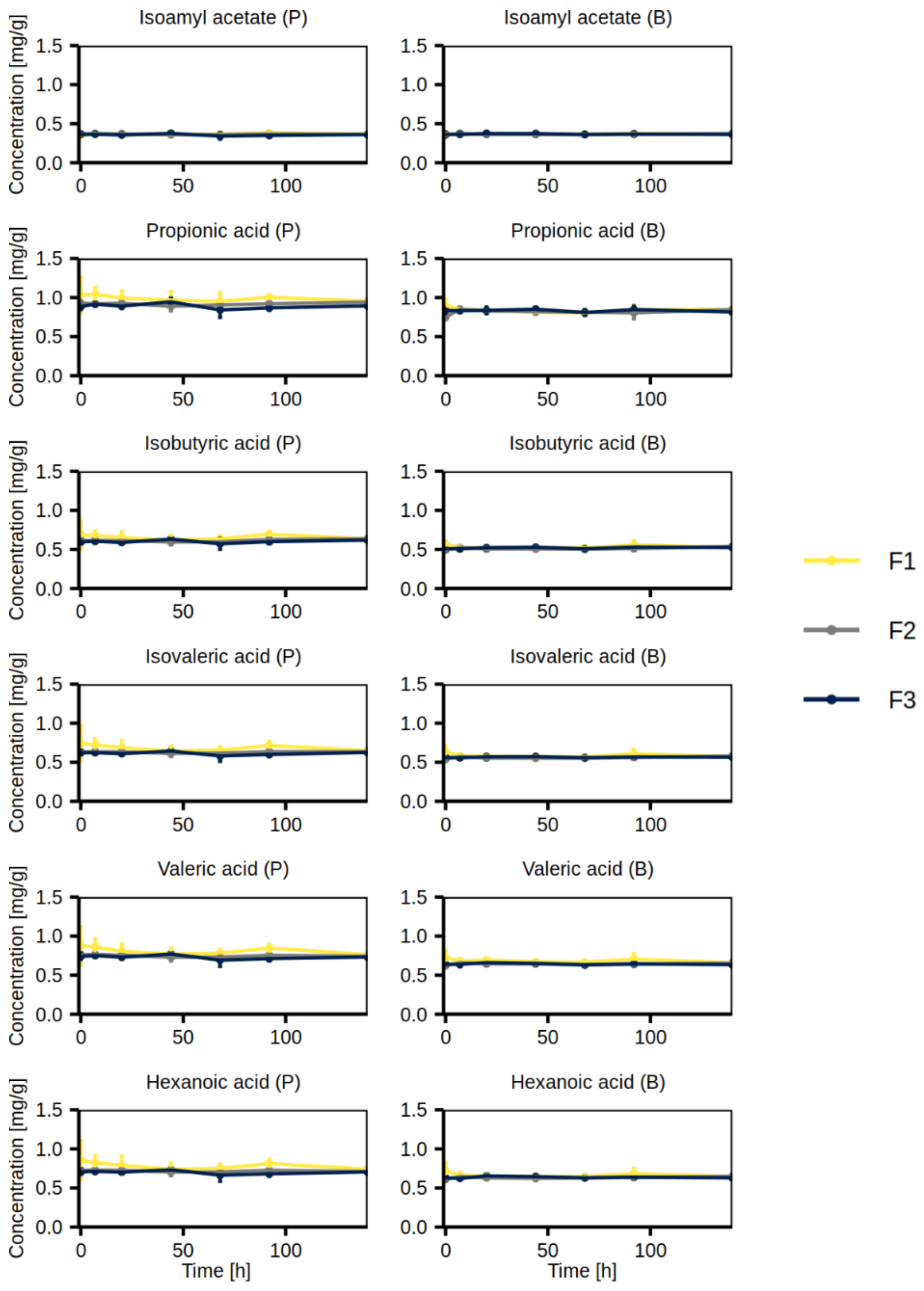

Supplement: Supplementary file 1 [file Data_Sheet_1.pdf]
