# Supplementary material for: A Combined Metagenomics and Metatranscriptomics Approach to Unravel Costa Rican Cocoa Box Fermentation Processes Reveals Yet Unreported Microbial Species and Functionalities
Source: Front Microbiol. 2021 Feb 16;12:641185. doi: 10.3389/fmicb.2021.641185 (PMC7920976; doi:10.3389/fmicb.2021.641185)
Supplement: Supplementary file 2 [file Data_Sheet_2.pdf]

Supplementary Table 1. Statistics of the quality-trimmed cocoa fermentation metagenomes of the Costa Rican cocoa box fermentation processes F1, F2, and F3.

Sample	Metagenome size [Mbp]	Number of reads	Median read length [bp]
CRF1D7	1037	4,031,745	266
CRF1D20	948	3,860,534	251
CRF1D68	759	3,789,985	203
CRF2D0	778	3,219,309	263
CRF2D7	691	2,942,932	258
CRF2D20	828	3,128,225	288
CRF2D44	820	3,135,287	285
CRF2D68	785	3,469,242	238
CRF2D92	812	3,248,160	278
CRF2D140	693	2,984,671	252
CRF3D8	998	4,068,362	260
CRF3D21	1063	4,037,582	278
CRF3D69	992	4,283,679	234

Supplementary Table 2. List of microbial species identified in the metagenomes of Costa Rican cocoa box fermentation processes F1, F2, and F3, ordered by decreasing maximum percentage of reads assigned to them or closely related species without an available genome sequence (*).

**Species**	**Maximum percentage of reads recruited**
Acetobacter pasteurianus	26.88
Limosilactobacillus fermentum	26.03
Liquorilactobacillus cacaonum	12.28
Hanseniaspora opuntiae	11.47
Lactiplantibacillus plantarum subsp. plantarum	10.85
Liquorilactobacillus nagelii	8.08
Phytophthora palmivora	7.71
Leuconostoc pseudomesenteroides	6.33
Acetobacter ghanensis	5.46
Paucilactobacillus vaccinostercus	5.15
Acetobacter ascendens	4.32
Saccharomyces cerevisiae	3.69
Levilactobacillus brevis	3.65
Lactiplantibacillus fabifermentans	3.45
Acetobacter pomorum	2.73
Acetobacter oryzifermentans	2.52
Lactiplantibacillus argentoratensis	2.24
**Cellvibrio japonicus*	1.84
Tatumella ptyseos	1.63
**Pectobacterium carotovorum*	1.50
Acetobacter fabarum	1.37
Candida tropicalis	1.34
Liquorilactobacillus mali	1.20
Liquorilactobacillus hordei	1.15
Acinetobacter populi	1.14
Komagataeibacter hansenii	0.97
Tatumella saanichensis	0.97
Lentilactobacillus raoultii	0.91
**Pectobacterium polaris*	0.87
Levilactobacillus spicheri	0.76
Gluconacetobacter entanii	0.71
**Apilactobacillus kunkeei*	0.66
Komagataeibacter maltaceti	0.62
Thielaviopsis ethacetica	0.62
Acetobacter okinawensis	0.59
Pichia manshurica	0.59
Lactiplantibacillus pentosus	0.53
Acetobacter papayae	0.52
Acetobacter indonesiensis	0.52

Supplementary Table 2. *(continued)*

Species	Maximum percentage of reads recruited
Liquorilactobacillus ghanensis	0.51
Secundilactobacillus collinoides	0.49
Dysgonomonas capnocytophagoides	0.48
Acetobacter tropicalis	0.42
Komagataeibacter saccharivorans	0.41
Torulaspora delbrueckii	0.41
Acetobacter persici	0.40
Erwinia toletana	0.38
Enterococcus italicus	0.38
Phytomonas serpens	0.37
Pichia occidentalis	0.37
Hanseniaspora pseudoguilliermondii	0.36
Gluconobacter japonicus	0.36
Frateuria aurantia	0.35
Komagataeibacter cocois	0.33
Acetobacter senegalensis	0.30
Komagataeibacter oboediens	0.25
Candida ethanolica	0.24
Fructobacillus pseudoficulneus	0.24
Komagataeibacter intermedius	0.24
Komagataeibacter nataicola	0.23
Candida orthopsilosis	0.21
Gluconobacter oxydans	0.21
Pantoea cypripedii	0.20
Pichia kluyveri	0.20
Lentilactobacillus farraginis	0.20
Fusarium metavorans	0.20
**Rhizobium smilacinae*	0.19
Fructobacillus tropaeoli	0.19
Erwinia iniecta	0.19
**Kosakonia oryzendophytica*	0.19
Komagataeibacter xylinus	0.18
Lactiplantibacillus mudanjiangensis	0.18
Paucilactobacillus suebicus	0.17
Candida sorboxylosa	0.17
Acetobacter orientalis	0.16
Acetobacter syzygii	0.15
Klebsiella variicola	0.15
Pantoea dispersa	0.15
Ketogulonicigenium robustum	0.14
Liquorilactobacillus satsumensis	0.14
Klebsiella oxytoca	0.13
Gluconobacter kondonii	0.13

Supplementary Table 2. *(continued)*

Species	Maximum percentage of reads recruited
Limosilactobacillus vaginalis	0.12
Candida stellimalicola	0.12
Sphingobacterium siyangense	0.11
Zymomonas mobilis	0.11
Pantoea rodasii	0.11
Citrobacter koseri	0.11
Acinetobacter gandensis	0.11
Moniliophthora roreri	0.10
Rhizobium larrymoorei	0.10
Hanseniaspora thailandica	0.09
Enterococcus casseliflavus	0.09
Gluconobacter sphaericus	0.09
Xanthomonas massiliensis	0.08
Bacteroides reticulotermitis	0.07
Acinetobacter qingfengensis	0.07
Lactococcus lactis	0.07
Lentilactobacillus hilgardii	0.07
Kosakonia oryzae	0.07
Colletotrichum fructicola	0.07
Pantoea coffeiphila	0.06
Lactobacillus amylovorus	0.06
Colletotrichum gloeosporioides	0.06
Weissella paramesenteroides	0.06
Microbacterium indicum	0.05
Weissella cibaria	0.05
Acinetobacter gerneri	0.05
Sphingobacterium wenxiniae	0.05
Providencia sneebia	0.05
Enterobacter roggenkampii	0.04
Enterobacter soli	0.04
Sphingobium yanoikuyae	0.04
Colletotrichum musae	0.04
Sphingobacterium paludis	0.04
Pichia kudriavzevii	0.03
Pantoea anthophila	0.03
Agrobacterium deltaense	0.03
